# An Updated Systematic Review and Meta‐Analysis on the Efficacy and Safety of Metformin as Add‐on Therapy to Insulin in Patients With Type 1 Diabetes

**DOI:** 10.1002/edm2.70060

**Published:** 2025-06-13

**Authors:** Mohammad Mahdi Masouri, Rasoul Ebrahimi, Shokoofe Noori

**Affiliations:** ^1^ School of Medicine Shahid Beheshti University of Medical Sciences Tehran Iran; ^2^ Department of Biochemistry Faculty of Medicine, Shahid Beheshti University of Medical Sciences Tehran Iran

**Keywords:** meta‐analysis, metformin, type 1 diabetes mellitus

## Abstract

**Introduction:**

This study aims to perform an updated meta‐analysis evaluating the efficacy and safety of metformin adjunct therapy in type 1 diabetes mellitus (T1DM) patients.

**Method:**

Cochrane, PubMed and Embase were searched for randomised controlled trials (RCTs) that reported the efficacy and safety of metformin in T1DM patients. Statistical analyses were performed using STATA software.

**Results:**

Twenty‐nine placebo‐controlled RCTs enrolling 2051 T1DM patients were included. Adolescents experienced a notable reduction in total insulin daily dose (TIDD) (mean difference [MD] = −0.61 [95% confidence interval (CI): −1.02, −0.20] units/kg per day) and levels of haemoglobin A1c (HbA1c) (MD = −0.45 [95% CI: −0.79, −0.11]), total cholesterol (TC) (MD = −0.78 [95% CI: −1.54, −0.02]), and low‐density lipoprotein (LDL) (MD = −0.69 [95% CI: −1.36, −0.02]) at 3 months of follow‐up with metformin. In adults, metformin significantly reduced Body Mass Index (BMI) (MD = −0.71 [95% CI: −1.23, −0.19]), TIDD (MD = −0.44 [95% CI: −0.73, −0.16]), and levels of HbA1c (MD = −0.70 [95% CI: −1.10, −0.30]) and TC (MD = −0.60 [95% CI: −1.09, −0.10]) at 6 months. The risk of gastrointestinal adverse events (GIAEs) was significantly higher in both adolescents (Relative Risk [RR] = 1.74 [95% CI: 1.38, 2.21]) and adults (RR = 3.24 [95% CI: 1.49, 7.02]). All of the above had *p*‐values less than 0.05. The metformin group showed no differences in BMI Z‐score, high‐density lipoprotein (HDL) level, or diabetic ketoacidosis (DKA) risk. No statistical difference was identified for any of the outcomes at other follow‐up endpoints.

**Conclusions:**

Metformin may reduce TIDD and levels of HbA1c, TC, triglycerides (TG), and LDL in T1DM adolescents. BMI, TIDD, and levels of HbA1c and TC may decrease in adults. Moreover, it may raise the risk of GIAEs in both age groups.

## Graphical abstract

This updated meta‐analysis evaluates the efficacy and safety of metformin as add‐on therapy to insulin in patients with type 1 diabetes. The results show reductions in HbA1c, BMI, and insulin dose in both adolescents and adults, alongside improved lipid profiles. However, metformin increased gastrointestinal adverse events, highlighting the need for further research on optimal dosing and duration.

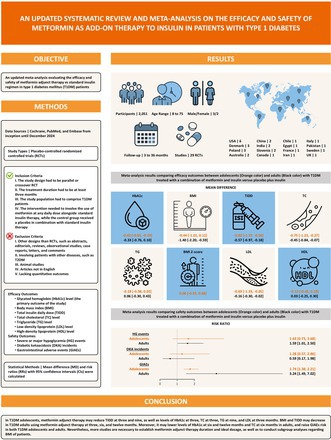

## 1 Introduction

Type 1 diabetes mellitus (T1DM) is an autoimmune disease driven by the infiltration of helper and cytotoxic T lymphocytes, along with macrophages, into the islets of Langerhans [1]. Typically, children diagnosed with T1DM show symptoms such as frequent urination, excessive thirst, and loss of weight, with around one‐third having diabetic ketoacidosis (DKA) [2]. The specific factors that trigger T1DM remain unclear, but there is an increasing consensus that it arises from an interplay of genetic predisposition and environmental influences. T1DM represents approximately 10% of all diabetes cases globally, with higher prevalence among individuals of European descent [1]. Research conducted by Gregory et al. indicated that in 2021, roughly 8.4 million people worldwide were living with T1DM, and estimates suggest this figure could rise to between 13.5 and 17.4 million by 2040 [3]. This expected surge in T1DM cases aligns with the notion that environmental factors contributing to genetic susceptibility are significant in understanding its epidemiology [1].

Over the past 30 years, some studies have demonstrated that intensive glycemic control can decrease the risk of microvascular‐ and cardiovascular‐related issues in T1DM [4, 5]. However, despite advancements in insulin formulations and delivery methods, many individuals still struggle to achieve and maintain target blood glucose levels [6]. A significant hindrance is the risk and anxiety surrounding hypoglycemia (HG), which increased sharply as glycated haemoglobin (HbA1c) levels neared target values in the DCCT [4]. While continuous subcutaneous insulin delivery has made this issue more manageable for motivated youth [7], other challenges include insulin‐related weight gain, which can lead to insulin resistance and higher insulin dosage requirements, along with rising low‐density lipoprotein (LDL) levels and blood pressure [8]. This situation has prompted the idea of adjunct therapy for T1DM, suggesting that an oral treatment alongside insulin could enhance glycemic control and potentially reduce the risk of complications independent of glucose lowering. Ideal adjunct treatments would decrease the need for insulin, lower HbA1c without increasing HG risk, reduce weight, and have additional benefits for cardiovascular health and life expectancy [9, 10].

In past years, clinicians have considered metformin as a potential adjunct therapy, reflecting its effects observed in type 2 diabetes mellitus (T2DM) when applied to T1DM. As an insulin‐sensitising agent, metformin improves insulin sensitivity and glucose uptake in T2DM [11, 12] while also being recognised as a recommended adjunct therapy in overweight T1DM patients, potentially reducing cardiovascular disease (CVD) risk [13, 14]. Up to now, several meta‐analyses examining the efficacy and safety of metformin adjunct therapy in T1DM patients have been conducted [15, 16, 17, 18, 19]. However, following the release of earlier meta‐analyses, some additional studies that could be eligible have been published [20, 21, 22, 23, 24, 25, 26, 27, 28]. To obtain more dependable findings that address conflicting outcomes, it is essential to integrate all available research. Thus, to provide a thorough evaluation of the efficacy and safety of metformin adjunct therapy in T1DM patients, this updated systematic review and meta‐analysis was carried out.

## 2 Materials and Methods

This study adheres to the guidelines specified in the Preferred Reporting Items for Systematic reviews and Meta‐Analyses (PRISMA) checklist [29].

### 2.1 Search Strategy

We searched the databases Cochrane, Embase, and PubMed until December 14, 2024, with no restrictions on time, study design, or language. We utilised a range of terms related to T1DM and Metformin: ((“Type 1 Diabetes”) OR (“Insulin‐Dependent Diabetes Mellitus”) OR (“Juvenile‐Onset Diabetes Mellitus”) OR (“IDDM”) OR (“Autoimmune Diabetes”)) AND (Metformin). The full search methodology is provided in Appendix S1. After reviewing the full texts, we assessed the first 300 results from Google Scholar up to December 17, 2024, which formed part of our search for grey literature [30]. We also conducted backward and forward citation searching.

### 2.2 Study Selection

Two independent authors (R.E. and M.M.M.) reviewed the titles and abstracts of the studies uploaded to Rayyan [31], an online tool designed to streamline the screening process, to determine their eligibility for inclusion in the study. To qualify for inclusion, studies needed to meet specific criteria, requiring: (a) the study design had to be RCT, whether parallel or crossover, without restrictions on geographical location and publication date; (b) the treatment duration had to be at least 3 months; (c) the study population had to comprise T1DM patients, with no sex or age limitations, who were actively undergoing insulin treatment, regardless of how long they had been diagnosed with diabetes; (d) the diagnosis of T1DM and the details regarding insulin administration, including dosage and regimen, had to be accepted based on standards of the trialists; (e) the intervention needed to involve the use of metformin at any daily dose alongside standard insulin therapy, while the control group received a placebo in combination with standard insulin therapy; (f) for a trial to be included, it was required to cover one or more of these outcomes: efficacy outcomes, which include changes from baseline in HbA1c level (%), which was the primary outcome of this systematic review and meta‐analysis, body mass index (BMI) in kg/m^2^ or as a *Z*‐score for age and gender, total insulin daily dose (TIDD) in units/kg, total cholesterol (TC) level in mmol/L, triglyceride (TG) level in mmol/L, LDL level in mmol/L, and/or high‐density lipoprotein (HDL) level in mmol/L; and safety outcomes, which cover the incidence of severe or major HG events, DKA incidents, and gastrointestinal adverse events (GIAEs). The criteria for exclusion were as specified: other designs than randomised controlled trials (RCTs), such as abstracts, editorials, reviews, observational studies, case reports, letters, and comments; involving patients with other diseases, such as T2DM; animal studies; articles not in English; and lacking quantitative outcomes. All studies that fulfilled the eligibility criteria were subjected to a full‐text review, which was carried out independently by the same two authors. Disputes were settled by consulting the principal investigator (SN).

### 2.3 Data Extraction

A template was prepared in Microsoft Excel Office 2021 for data extraction. RE and MMM carried out the data extraction separately, while SN addressed any disputes or disagreements that arose. The baseline study characteristics collected comprised: first writer, year of study publication, the country where the study was conducted, the design of the study, population, follow‐up duration, intervention and dose, weight, number of patients, number of males, number of patients who did not complete follow‐up, age, duration of T1DM, efficacy outcomes, safety outcomes, and trial registration.

### 2.4 Quality Assessment

To assess the risk of bias, the Revised Cochrane risk‐of‐bias tool for randomised trials (RoB 2) was utilised [32], focusing on seven specific items: generation of random sequences, concealment of allocations, patients and staff blindness, outcome assessor blindness, handling of data gaps, selective disclosure and other possible bias sources. Every criterion was scored as ‘low risk of bias’, ‘some concerns or moderate risk of bias’, or ‘high risk of bias’. Any disputes were settled through discussion the principal investigator (S.N.).

### 2.5 Statistical Analysis

Continuous variables findings were expressed using the mean difference (MD), while the risk ratio (RR) was employed for dichotomous variables, both accompanied by their respective 95% confidence intervals (CIs). The median, interquartile range, minimum, and maximum values were converted to mean and standard deviation [33, 34]. Changes from baseline (delta) were calculated for each group (intervention and control separately). The pooled standard deviation was then computed. Additionally, the conversion of lipid values from mg/dL to mmol/L was performed. We assessed the degree of heterogeneity utilising the *I* ^2^ statistic [35]. A random‐effects model was used to calculate pooled results when heterogeneity was deemed significant, indicated by an *I* ^2^ exceeding 50% [35]. Conversely, if the *I* ^2^ value was below 50%, we employed fixed‐effects models for the meta‐analysis [36]. Subgroup analyses were also carried out to examine the effects of metformin on BMI, BMI *Z*‐scores, TIDD, and levels of HbA1c, TC, TG, LDL, and HDL, with the data being reported at various endpoints including three, 6, 9, 12, and 36 months of follow‐up. Additionally, separate analyses were performed based on age groups (adults or adolescents). To qualitatively examine publication bias, a funnel plot was used [37]. To evaluate the symmetry of a funnel plot, Egger's and Begg's tests were conducted [38]. Two‐sided *p*‐values were calculated, and a *p* < 0.05 was considered significant. Utilising STATA (version 18), the meta‐analysis was carried out.

## 3 Results

**FIGURE 1:**
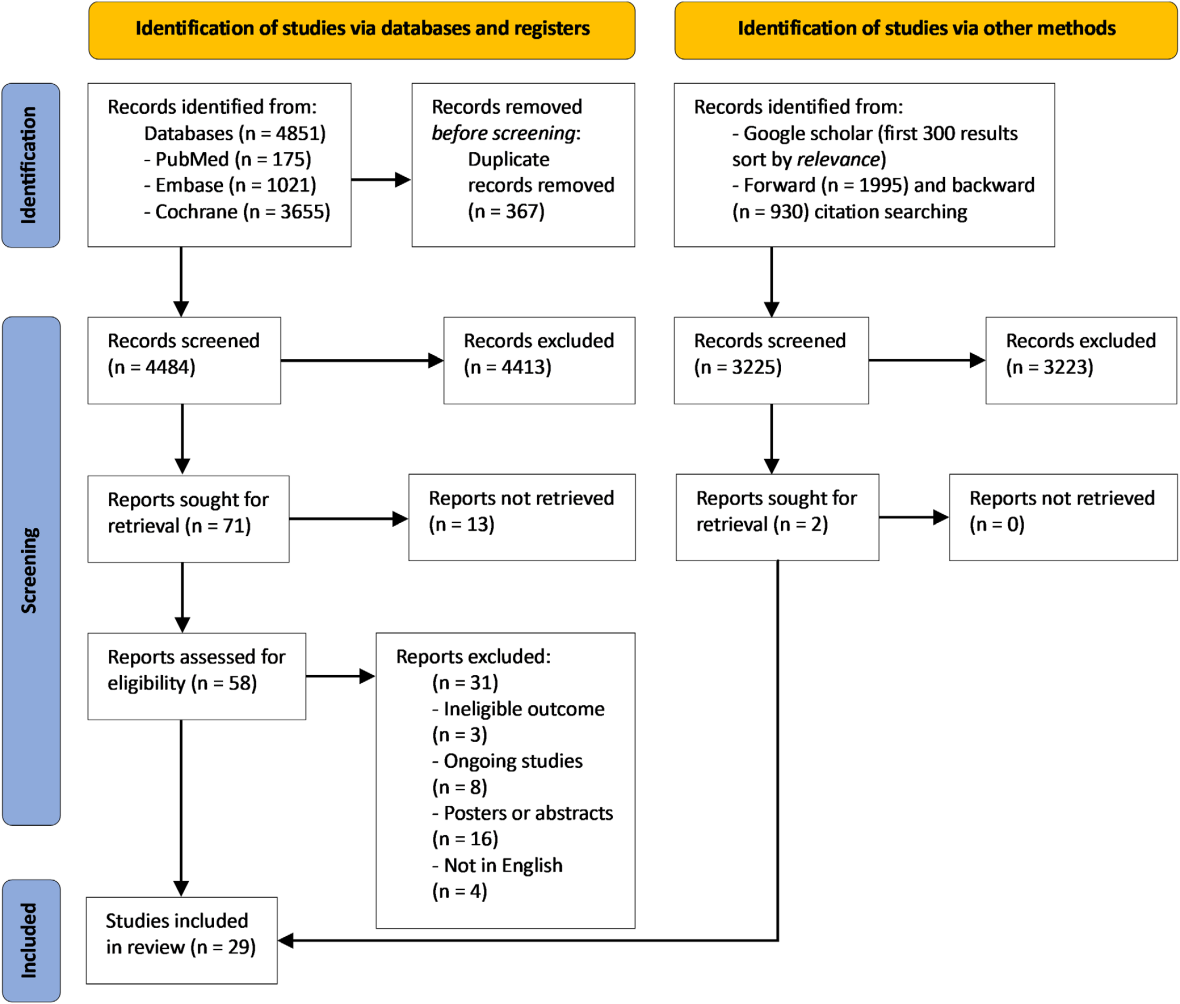
PRISMA flowchart. Study selection process for the articles in the systematic review and meta‐analysis.

The systematic search yielded 4851 articles. Once duplicates were removed, 4484 articles were assessed by their titles and abstracts, leading to the exclusion of 4413 articles. Of the 71 articles left, 13 were excluded after further evaluation, and 58 were selected for full‐text review. These articles were then subjected to full‐text review. Three articles were excluded for not reporting the required outcomes, 8 were ongoing studies, 16 were posters or abstracts, and 4 were not written in English. Thus, 27 studies were included at this stage. Two additional studies were found through citation and Google Scholar searching. A total of 29 placebo‐controlled RCTs were incorporated into the meta‐analysis [14, 20, 21, 22, 23, 24, 25, 26, 27, 28, 39, 40, 41, 42, 43, 44, 45, 46, 47, 48, 49, 50, 51, 52, 53, 54, 55, 56, 57]. Figure 1 shows the PRISMA flowchart in detail.

### 3.1 Study Characteristics

Appendix S2 outlines the study design and baseline characteristics of the identified RCTs. Six RCTs were carried out in the United States of America [40, 44, 45, 49, 54, 55], three in each Denmark [47, 50, 51] and Poland [27, 41, 42], two in each Australia [14, 39], China [26, 28], India [23, 24], and Slovenia [22, 52], and one in each Canada [46], Chile [43], Egypt [21], France [53], Iran [25], Italy [56], Pakistan [20], Sweden [57] and the United Kingdom [48]. The total sample size was 2051, with ages ranging from about 8 [39] to 75 [28] years. The male/female ratio was about 3/2 totally and T1DM duration was at least between about 6 months [39] and 5 years [41].

### 3.2 Meta‐Analysis

A summary of the MD and RR subgroup analysis is presented in Appendix S3.

### 3.3 Risk of Bias

Overall, a moderate overall risk of bias was assessed for seven RCTs [20, 26, 27, 28, 41, 42, 46], and another 22 RCTs were deemed to have a low overall risk of bias [14, 21, 22, 23, 24, 25, 39, 40, 43, 44, 45, 47, 48, 49, 50, 51, 52, 53, 54, 55, 56, 57]. Of these 29 RCTs, all had an appropriate randomization process. Both crossover RCTs [26, 48] had a sufficient time for carryover effects. All but three [26, 27, 46] RCTs applied blinding to staff, outcome assessors, and patients. All RCTs had data available for all patients. All but five [20, 26, 28, 41, 42] RCTs had an appropriate method of outcome measurement, and all RCTs had a prespecified analysis plan (Appendices S4 and S5).

### 3.4 Body Mass Index

**FIGURE 2:**
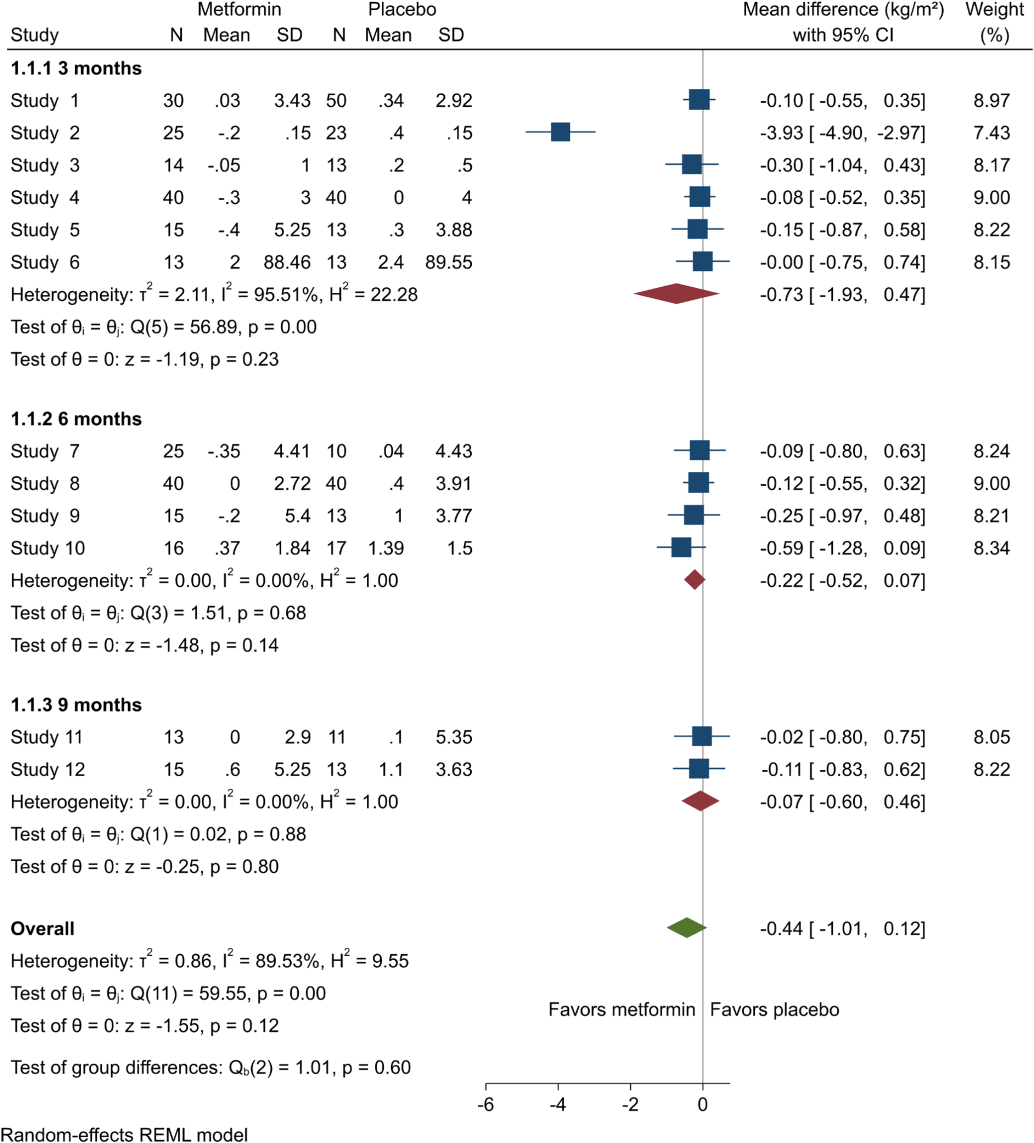
Forest plots showing the BMI of adolescents. Forest plots illustrating the meta‐analysis results comparing BMI between adolescents with T1DM treated with a combination of metformin and insulin versus placebo plus insulin.

**FIGURE 3:**
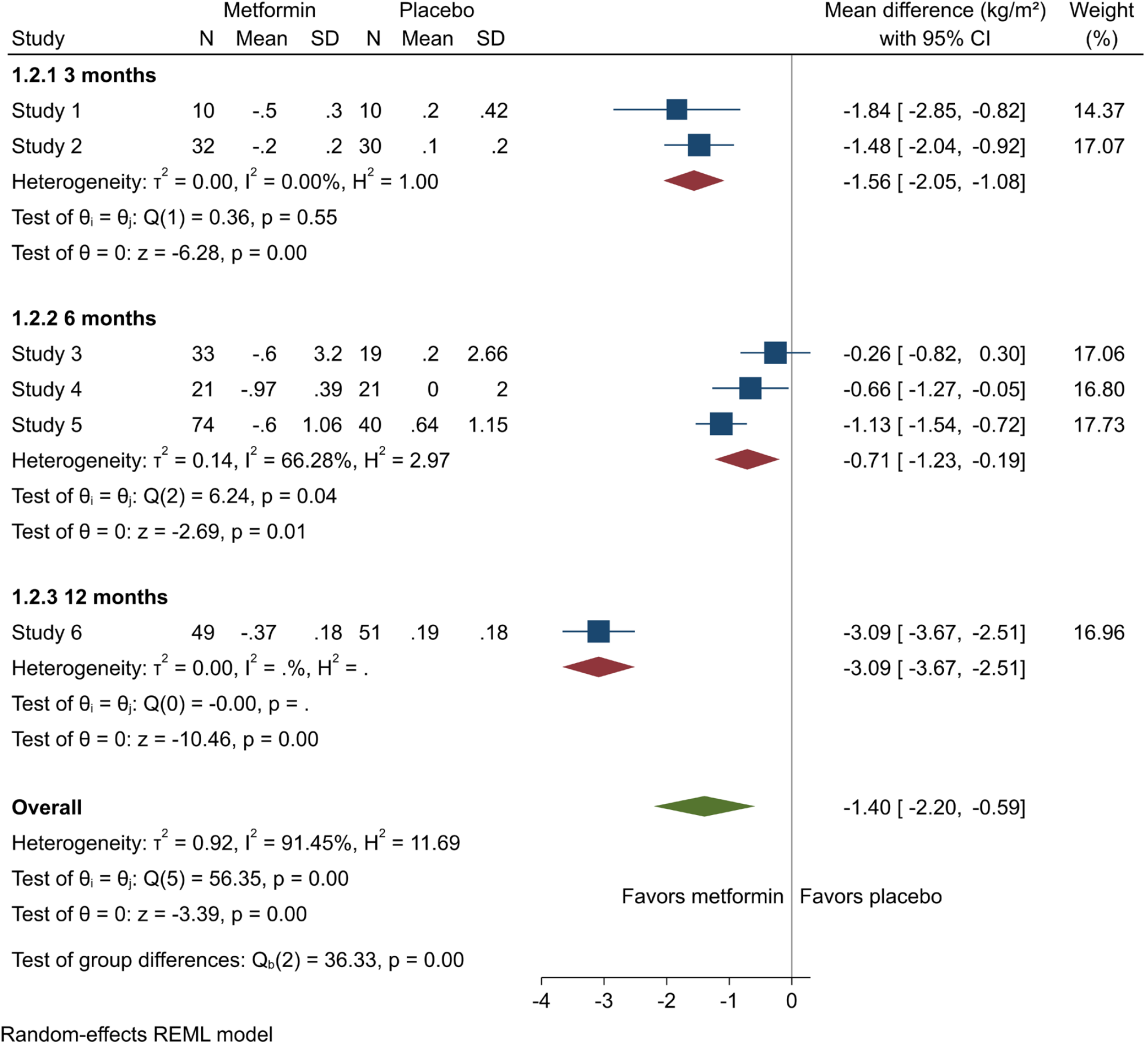
Forest plots showing the BMI of adults. Forest plots illustrating the meta‐analysis results comparing BMI between adults with T1DM treated with a combination of metformin and insulin versus placebo plus insulin.

BMI and BMI Z‐score data were provided by 15 [20, 26, 27, 28, 40, 42, 43, 45, 46, 50, 52, 54, 55, 56, 57] and eight [21, 23, 24, 40, 44, 45, 49, 54] studies, respectively. In adolescents, the changes from baseline in BMI showed no significant improvements at any follow‐up endpoints, whether at three (MD = −0.73 [95% CI: −1.93, 0.47], *p* = 0.23, *I* ^2^ = 95.51%), six (MD = −0.22 [95% CI: −0.52, 0.07], *p* = 0.14, *I* ^2^ = 0.00%), or nine (MD = −0.07 [95% CI: −0.60, 0.46], *p* = 0.80, *I* ^2^ = 0.00%) months in the metformin group compared to the control group (Figure 2). Metformin significantly reduced BMI among adults at three (MD = −1.56 [95% CI: −2.05, −1.08], *p* = 0.00, *I* ^2^ = 0.00%) and six (MD = −0.71 [95% CI: −1.23, −0.19], *p* = 0.01, *I* ^2^ = 66.28%) months (Figure 3). BMI *Z*‐score among adolescents also showed no notable difference at three (MD = 0.41 [95% CI: −0.72, 1.55], *p* = 0.48, *I* ^2^ = 96.94%) or six (MD = −0.31 [95% CI: −0.70, 0.08], *p* = 0.12, *I* ^2^ = 64.94%) months (Appendix S6).

### 3.5 Total insulin daily dose

**FIGURE 4:**
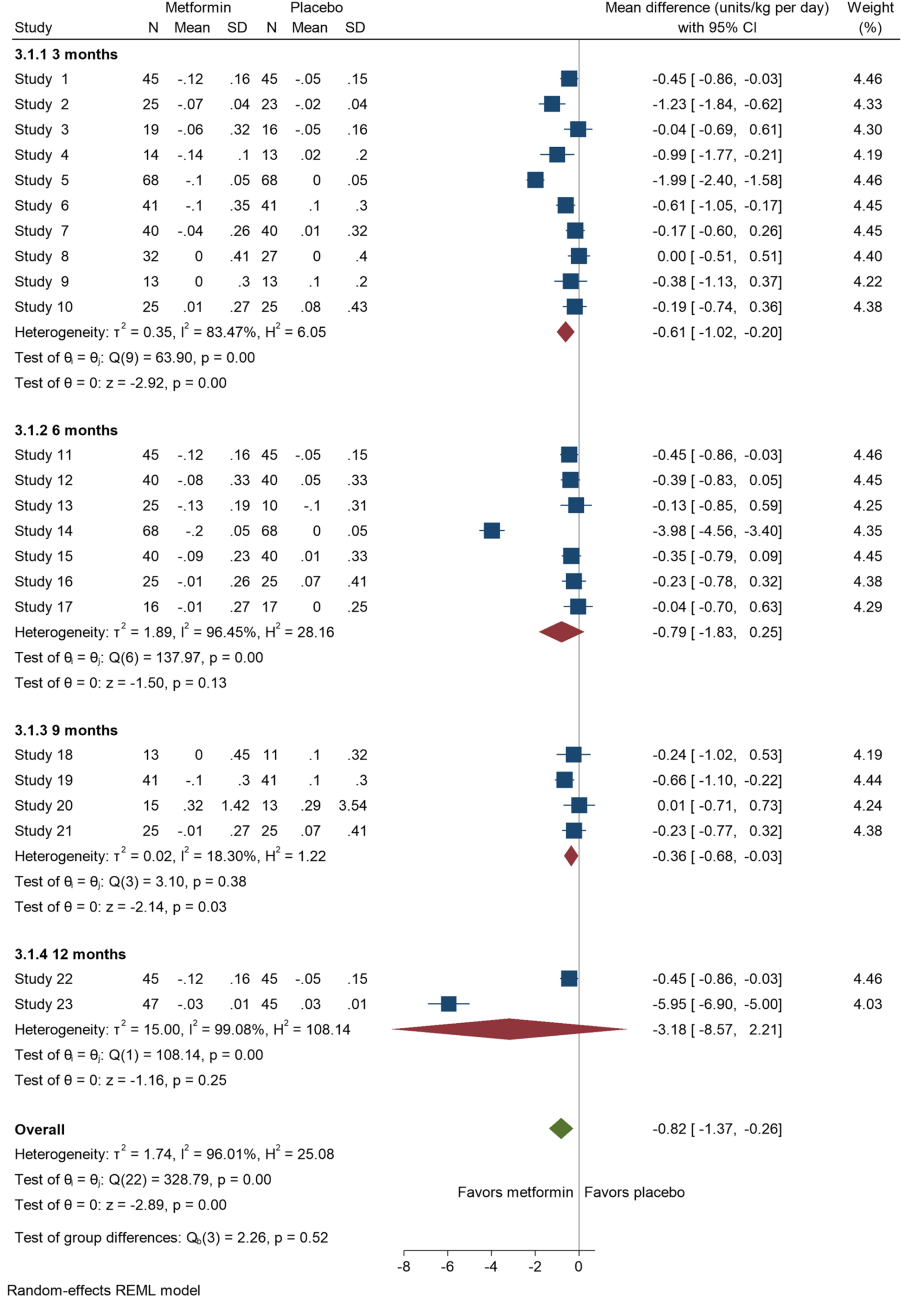
Forest plots showing the TIDD of adolescents. Forest plots illustrating the meta‐analysis results comparing TIDD between adolescents with T1DM treated with a combination of metformin and insulin versus placebo plus insulin.

**FIGURE 5:**
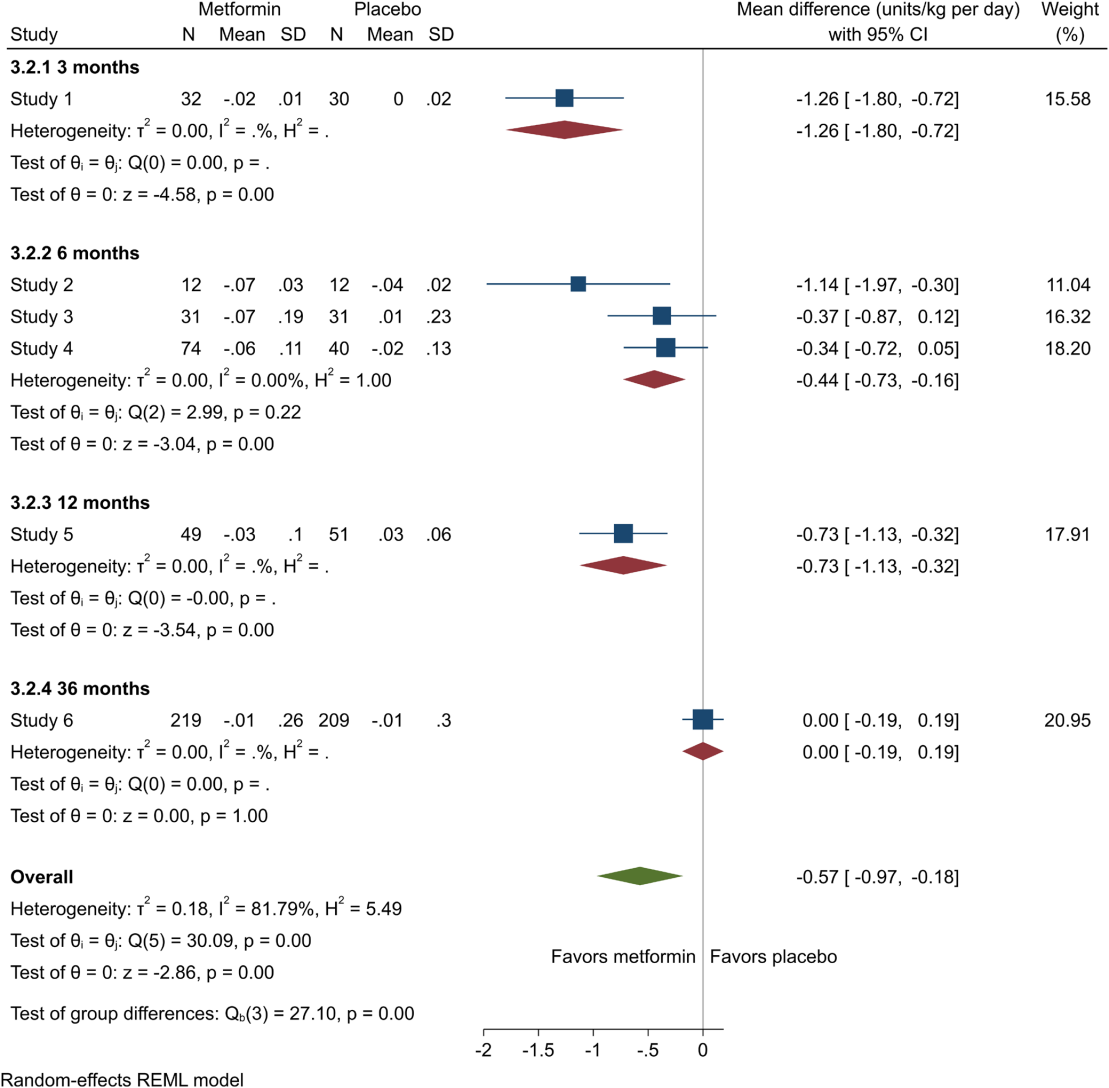
Forest plots showing the TIDD of adults. Forest plots illustrating the meta‐analysis results comparing TIDD between adults with T1DM treated with a combination of metformin and insulin versus placebo plus insulin.

Data on TIDD was contributed by 21 studies [14, 21, 23, 24, 25, 26, 27, 28, 39, 40, 43, 44, 45, 46, 47, 49, 50, 51, 53, 54, 57]. There was a significant decrease in TIDD at three (MD = −0.61 [95% CI: −1.02, −0.20], *p* = 0.00, *I* ^2^ = 83.47%) and nine (MD = −0.36 [95% CI: −0.68, 0.03], *p* = 0.03, *I* ^2^ = 18.30%) months. At 6 and 12 months, the results, although not statistically significant, showed a decrease in TIDD (6 months: MD = −0.79 [95% CI: −1.83, 0.25], *p* = 0.13, *I* ^2^ = 96.45%; 12 months: MD = −3.18 [95% CI: −8.57, 2.21], *p* = 0.25, *I* ^2^ = 99.08%) (Figure 4). For adults, metformin significantly reduced TIDD at 6 months (MD = −0.44 [95% CI: −0.73, −0.16], *p* = 0.00, *I* ^2^ = 0.00%) (Figure 5).

### 3.6 HbA1c Level

**FIGURE 6:**
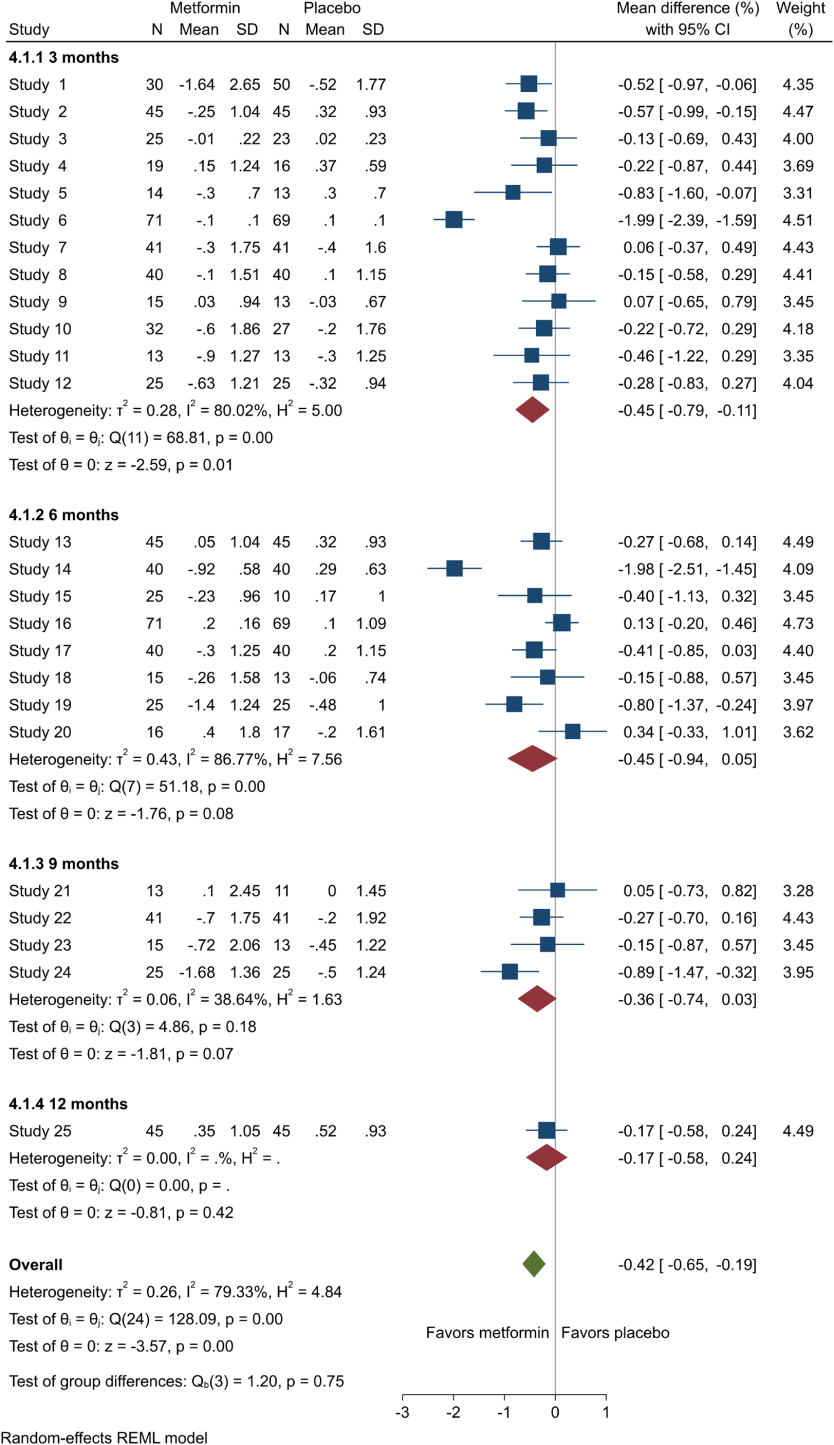
Forest plots showing the HbA1c of adolescents. Forest plots illustrating the meta‐analysis results comparing HbA1c between adolescents with T1DM treated with a combination of metformin and insulin versus placebo plus insulin.

**FIGURE 7:**
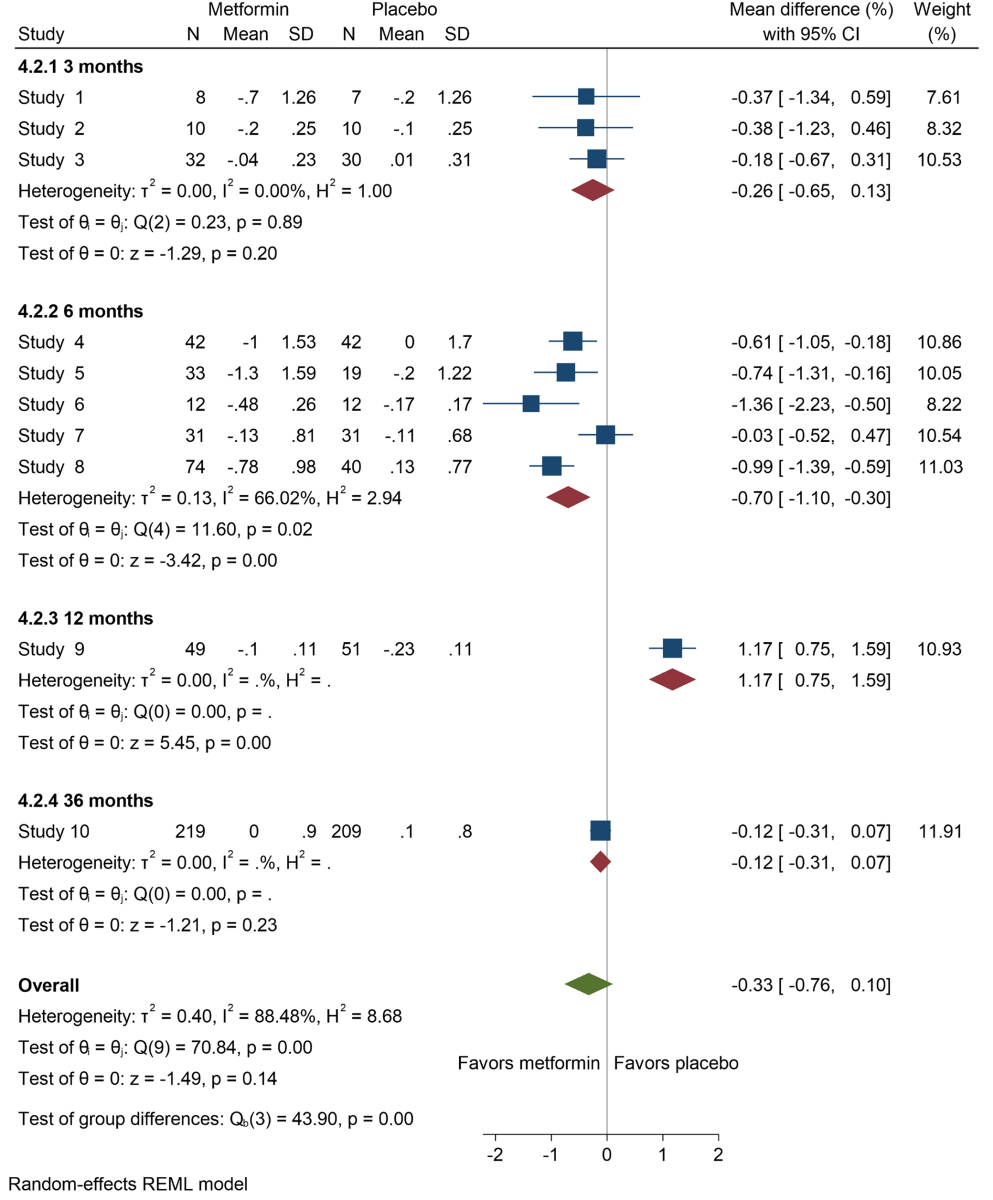
Forest plots showing the HbA1c level of adults. Forest plots illustrating the meta‐analysis results comparing HbA1c level between adults with T1DM treated with a combination of metformin and insulin versus placebo plus insulin.

Data on HbA1c level was obtained from 26 studies [14, 20, 21, 23, 24, 25, 26, 27, 28, 39, 40, 41, 42, 43, 44, 45, 46, 47, 48, 49, 50, 52, 53, 54, 55, 57]. Our results showed metformin significantly reduced HbA1c level for adolescents at 3 months (MD = −0.45 [95% CI: −0.79, −0.11], *p* = 0.01, *I* ^2^ = 80.02%). However, at 6 months, the reduction was not notable (MD = −0.45 [95% CI: −0.94, 0.05], *p* = 0.08, *I* ^2^ = 86.77%), nor was it at 9 months (MD = −0.36 [95% CI: −0.74, 0.03], *p* = 0.07, *I* ^2^ = 38.64%) (Figure 6). For adults, HbA1c level showed no significant difference at 3 months (MD = −0.26 [95% CI: −0.65, 0.13], *p* = 0.20, *I* ^2^ = 0.00%), but a significant reduction was observed at 6 months (MD = −0.70 [95% CI: −1.10, −0.30], *p* = 0.00, *I* ^2^ = 66.02%) (Figure 7).

### 3.7 TC Level

Seventeen studies [21, 23, 24, 25, 26, 27, 28, 39, 40, 42, 45, 47, 48, 49, 51, 53, 54] provided TC level data. For adolescents, the results at 3 months indicated a notable decrease in TC level (MD = −0.78 [95% CI: −1.54, −0.02], *p* = 0.04, *I* ^2^ = 92.46%). However, at 6 months, the difference was not statistically notable (MD = −0.47 [95% CI: −1.14, 0.21], *p* = 0.18, *I* ^2^ = 91.29%), and similar findings were observed at nine (MD = −0.67 [95% CI: −1.37, 0.02], *p* = 0.06, *I* ^2^ = 72.88%) and 12 (MD = −1.63 [95% CI: −4.49, 1.22], *p* = 0.26, *I* ^2^ = 98.38%) months (Appendix S7). In adults, the change in TC level was not significant at 3 months (MD = −0.09 [95% CI: −0.53, 0.34], *p* = 0.68, *I* ^2^ = 0.00%), but at 6 months, there was a statistically notable reduction in TC level (MD = −0.60 [95% CI: −1.09, −0.10], *p* = 0.02, *I* ^2^ = 69.20%) (Appendix S8).

### 3.8 TG Level

TG level data was provided by 17 studies [21, 23, 24, 25, 26, 27, 28, 39, 40, 41, 42, 45, 47, 48, 49, 51, 53]. In adolescents, TG level at three (MD = −0.27 [95% CI: −0.68, 0.14], *p* = 0.20, *I* ^2^ = 76.54%), six (MD = −0.03 [95% CI: −0.45, 0.40], *p* = 0.90, *I* ^2^ = 73.53%), and 12 (MD = 0.01 [95% CI: −0.28, 0.30], *p* = 0.94, *I* ^2^ = 0.00%) months showed no statistically significant reduction. However, at 9 months, metformin significantly reduced TG level (MD = −0.47 [95% CI: −0.81, −0.13], *p* = 0.01, *I* ^2^ = 0.00%) (Appendix S9). In adults, assessing the change in TG level at three (MD = −0.18 [95% CI: −0.62, 0.26], *p* = 0.42, *I* ^2^ = 0.00%) and six (MD = 0.19 [95% CI: −0.36, 0.73], *p* = 0.50, *I* ^2^ = 82.53%) months exhibited no notable changes (Appendix S10).

### 3.9 LDL Level

LDL level data was provided by 16 studies [14, 23, 24, 25, 26, 27, 28, 39, 40, 41, 42, 45, 47, 48, 49, 51]. For adolescents, metformin significantly reduced LDL level at 3 months (MD = −0.69 [95% CI: −1.36, −0.02], *p* = 0.04, *I* ^2^ = 90.69%). Conversely, no significant improvements were observed at six (MD = −0.55 [95% CI: −1.98, 0.88], *p* = 0.45 *I* ^2^ = 96.41%), nine (MD = −0.06 [95% CI: −0.56, 0.68], *p* = 0.85, *I* ^2^ = 68.63%), and 12 (MD = −1.74 [95% CI: −4.73, 1.24], *p* = 0.25, *I* ^2^ = 98.45%) months (Appendix S11). In adults, the impact of metformin on LDL level was not statistically significant at both three (MD = −0.21 [95% CI: −0.65, 0.22], *p* = 0.34, *I* ^2^ = 0.00%) and six (MD = −0.14 [95% CI: −0.38, 0.10], *p* = 0.25, *I* ^2^ = 23.96%) months (Appendix S12).

### 3.10 HDL Level

Sixteen studies provided HDL level data [23, 24, 25, 26, 27, 28, 39, 40, 41, 42, 45, 47, 48, 49, 51, 54]. For adolescents, at none of the time points, including three (MD = 0.18 [95% CI: −0.24, 0.61], *p* = 0.40, *I* ^2^ = 68.46%), six (MD = −0.16 [95% CI: −0.62, 0.31], *p* = 0.51, *I* ^2^ = 77.96%), nine (MD = −0.14 [95% CI: −0.52, 0.24], *p* = 0.47, *I* ^2^ = 17.50%), and 12 (MD = −0.74 [95% CI: −2.20, 0.72], *p* = 0.32, *I* ^2^ = 95.58%) months, there was a notable change from baseline in HDL level (Appendix S13). For adults, at three (MD = 0.14 [95% CI: −0.29, 0.58], *p* = 0.52, *I* ^2^ = 0.00%) and six (MD = 0.04 [95% CI: −0.21, 0.28], *p* = 0.76, *I* ^2^ = 68.01%) months, there were also no significant improvements (Appendix S14).

### 3.11 Adverse Events

**FIGURE 8:**
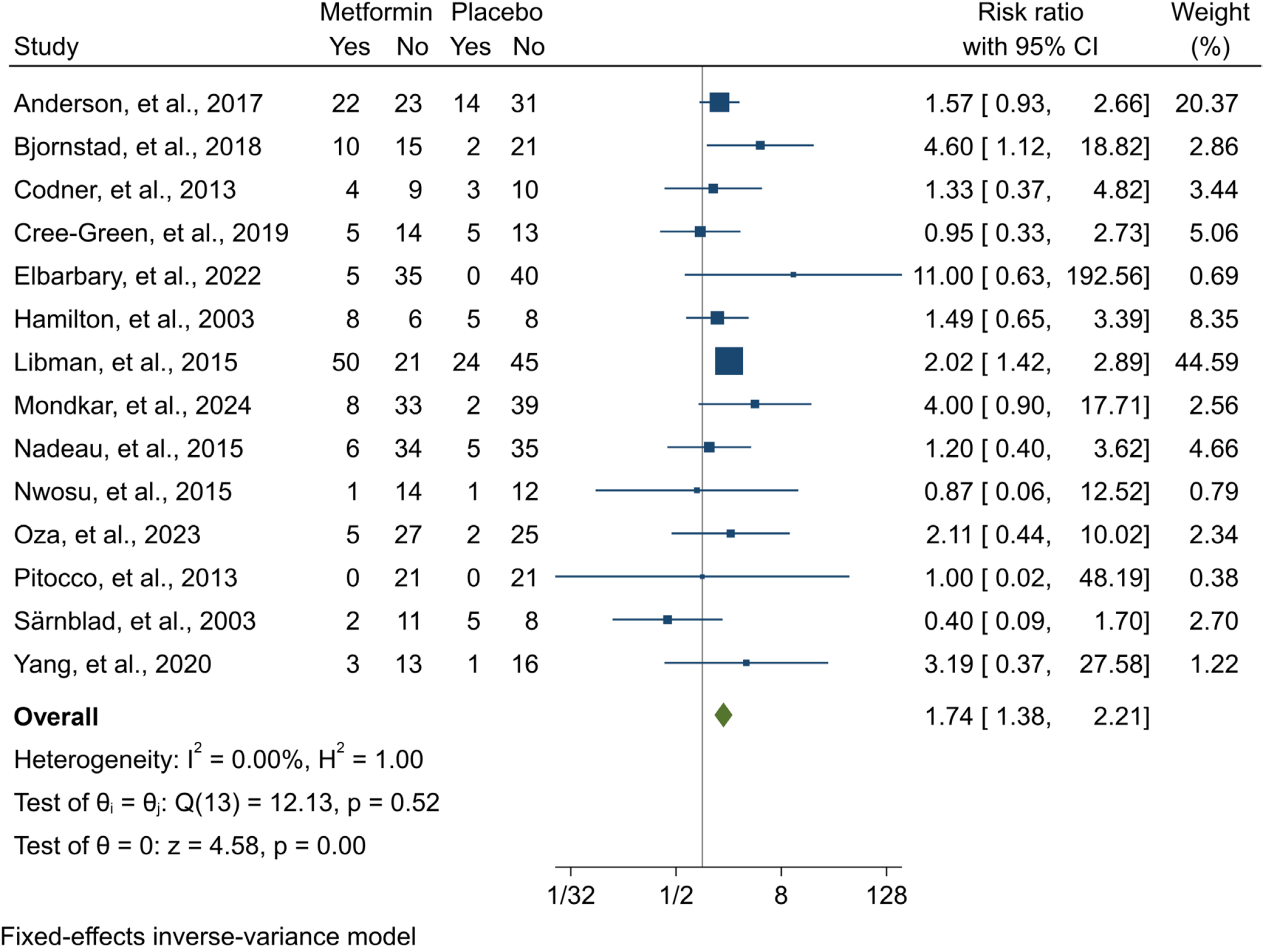
Forest plot showing the GIAEs risk of adolescents. Forest plot illustrating the meta‐analysis results comparing the risk of GIAEs between adolescents with T1DM treated with a combination of metformin and insulin versus placebo plus insulin.

**FIGURE 9:**
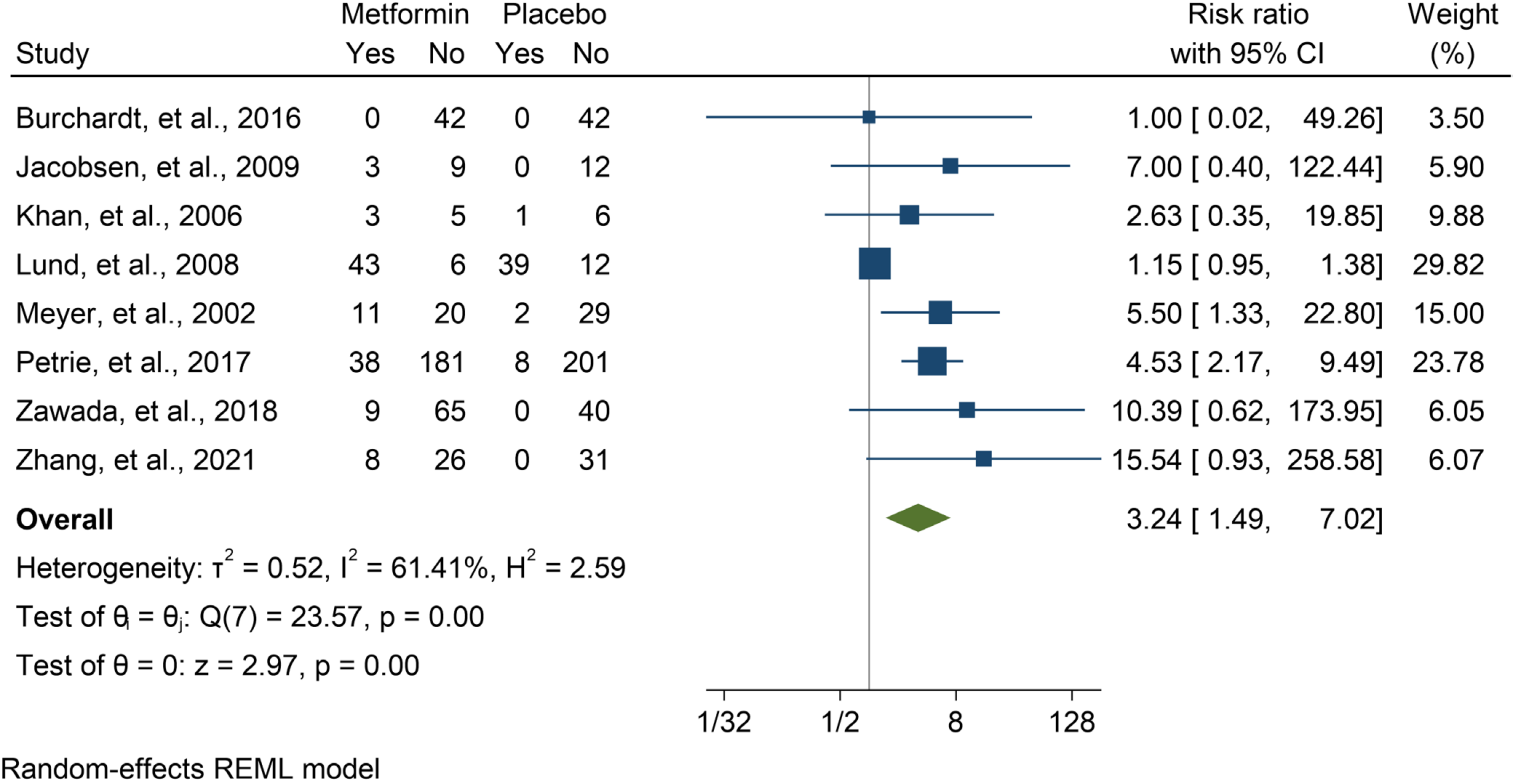
Forest plot showing the GIAEs risk of adults. Forest plot illustrating the meta‐analysis results comparing the risk of GIAEs between adults with T1DM treated with a combination of metformin and insulin versus placebo plus insulin.

Data on DKA, GIAEs, and HG was provided by 24 [14, 21, 22, 23, 24, 25, 26, 27, 28, 39, 40, 41, 43, 44, 46, 47, 48, 49, 50, 53, 54, 55, 56, 57], 22 [14, 21, 23, 24, 26, 27, 28, 39, 40, 41, 43, 44, 46, 47, 48, 49, 50, 53, 54, 55, 56, 57], and 22 [21, 22, 23, 24, 25, 26, 27, 28, 39, 40, 41, 43, 44, 46, 48, 49, 50, 53, 54, 55, 56, 57] studies, respectively. For DKA, there was no significant increased risk in adolescents (RR = 1.28 [95% CI: 0.57, 2.86], *p* = 0.55, *I* ^2^ = 0.00%) (Appendix S15) and adults (RR = 0.59 [95% CI: 0.17, 1.98], *p* = 0.39 *I* ^2^ = 0.00%) (Appendix S16). Regarding GIAEs, both adolescents (RR = 1.74 [95% CI: 1.38, 2.21], *p* = 0.00, *I* ^2^ = 0.00%) (Figure 8) and adults (RR = 3.24 [95% CI: 1.49, 7.02], *p* = 0.00, *I* ^2^ = 61.41%) (Figure 9) had a significant increased risk. Finally, for HG, adolescents had an insignificant increased risk (RR = 1.63 [95% CI: 0.73, 3.68], *p* = 0.24, *I* ^2^ = 0.00%) (Appendix S17). In contrast, adults had a higher risk of HG with metformin use (RR = 1.59 [95% CI: 1.01, 2.50], *p* = 0.05 *I* ^2^ = 44.88%) (Appendix S18).

### 3.12 Publication Bias

The existence of publication bias in BMI was confirmed by Egger's and Begg's tests (Egger's test: 0.02, Begg's test: 0.04) and HDL level for adolescents (Egger's test: 0.03, Begg's test: 0.05). BMI *Z*‐score for adolescents (Egger's test: 0.01, Begg's test: 0.38), TIDD for adults (Egger's test: 0.00, Begg's test: 0.13), and TG level for adults (Egger's test: 0.00, Begg's test: 0.07) had significant Egger's test but insignificant Begg's test. In other analyses, neither Egger's nor Begg's tests were significant (Appendix S3). The funnel plots are available in Appendices S19 and S20.

## 4 Discussion

We found that in adolescents, metformin did not significantly improve BMI, BMI *Z*‐score and HDL level at any follow‐up endpoints, but demonstrated some efficacy in reducing TIDD (at three and 9 months of follow‐up) and levels of HbA1c (at 3 months), TC (at 3 months), TG (at 9 months), and LDL (at 3 months). In adults, significant reductions in BMI (at any follow‐up endpoints including 3, 6, and 12 months), TIDD (at 3, 6, and 12 months), and levels of HbA1c (at 6 and 12 months) and TC (at 6 months) were observed, but metformin did not significantly reduce levels of TG, LDL, and HDL at any follow‐up endpoints. Side effects were also noted. In our meta‐analysis, we observed a significant increase in the risk of GIAEs with metformin in both adolescents and adults, which indicates a clear association between metformin use and a higher risk of GIAEs in these populations, with adults showing a notably higher risk. When comparing our results to previous studies, the findings align closely with those of Liu et al. [58], who reported an increased risk of GIAEs with metformin (RR = 1.38, 95% CI: 1.10–1.74, *p* = 0.005) in patients with T1DM. Similarly, Liu et al. [19] found a significant increase in GIAEs in adolescents (RR = 1.64, 95% CI: 1.28–2.10, *p* < 0.01), which is consistent with our findings in this population. However, Xu et al. [59] found an increased number of gastrointestinal events in the metformin group, but this difference was not statistically significant (MD = 1.59, *p* = 0.39).

In adults, the observed increase in HG risk with metformin use as an add‐on therapy to insulin is of particular clinical interest. While this finding approached statistical significance, it is important to consider that the increased risk of HG was driven largely by one study, while others reported wide confidence intervals. This highlights the complexity of interpreting HG outcomes, as the potential increased risk may not be consistent. The variation in study findings suggests that factors such as baseline insulin dosages, patient characteristics, and the presence of other comorbidities may influence the likelihood of HG events. From a clinical standpoint, while the overall risk of HG with metformin in adults remains relatively low, the increased risk identified in some studies calls for heightened vigilance, especially in patients who are already at higher risk of HG, such as those with poorly controlled diabetes or those requiring high doses of insulin. It is important for clinicians to carefully consider these factors when adding metformin to insulin therapy.

Moreover, in adolescents, metformin led to a reduction in HbA1c of −0.45% at 3 months, which, while statistically significant (*p* = 0.01), is often considered clinically modest. In many clinical settings, changes in HbA1c of less than 0.5% are considered unlikely to translate into meaningful improvements in long‐term outcomes such as microvascular or macrovascular complications, overall quality of life, potential side effects, cost‐effectiveness, and the patient's overall treatment goals. This raises an important point about the clinical relevance of our findings. Clinicians should carefully evaluate the overall benefit–risk profile when considering metformin as an adjunctive treatment in T1DM, particularly in populations where more substantial improvements in glycemic control are needed.

Metformin is a widely used pharmaceutical agent for managing T2DM, primarily functioning by inhibiting mitochondrial respiratory chain complex‐1, which affects intracellular energy and activates AMP‐activated protein kinase (AMPK) [60]. This activation plays a crucial role in decreasing hepatic gluconeogenesis and glucose output while enhancing glucose uptake in peripheral tissues. Additionally, metformin enhances the release of glucagon‐like peptide‐1 (GLP‐1), contributing to better glycemic control [60]. Emerging evidence suggests that metformin also alters the composition of intestinal microbiota, contributing to its antihyperglycemic effects [61]. Beyond its glucose‐lowering capabilities, metformin exerts pleiotropic effects through AMPK activation, including enhancement of endothelial function, suppression of proinflammatory pathways in adipose tissue, and reduced fatty acid oxidation and lipid levels. It additionally prevents the creation of advanced glycation end products (AGEs) by inactivating methylglyoxal independently of AMPK, potentially providing cardioprotection [10]. Clinical studies have shown that metformin treatment results in reduced fasting plasma glucose (FPG), HbA1c, and various lipid levels including LDL and TG, alongside a mild or weight‐neutral impact on body mass in patients with T2DM. When combined with insulin therapy, metformin can optimise glycemic control, diminish insulin dosage, prevent insulin‐induced weight gain, and lower the risk of complications. Notably, metformin is well regarded for its safety and tolerability, reinforcing its status as a cornerstone in the management of T2DM [62].

The prominence of metformin escalated following the UK Prospective Diabetes Study (UKPDS) published in 1998, which demonstrated its efficacy, particularly for obese individuals [63]. While initially approached with caution due to concerns over lactic acidosis—similar to the withdrawn biguanide phenformin—metformin gained acceptance after UKPDS revealed that patients using it experienced less weight gain, lower HG event rates, and a notable 33% reduction in myocardial infarction risk [63, 64]. In the 1980s, early research began to study the effects of metformin in T1DM patients. In the mid‐1980s, a small controlled trial carried out in France showed that adding metformin to insulin therapy for 7 days improved insulin sensitivity, as assessed by the euglycemic‐hyperinsulinemic clamp method, in 10 non‐obese individuals with T1DM [65]. Two years later, in 1987, the late Harry Keen presented findings at the EASD Annual Meeting from another double‐blind, placebo‐controlled crossover trial involving eight participants with T1DM. This 3‐week study found no significant changes in FPG levels, body weight, or insulin dosage requirements with metformin, although there was a notable improvement in the seven‐point capillary glucose profile [66].

Due to the strong evidence supporting its efficacy in T2DM, the off‐label use of metformin for treating T1DM has become quite prevalent in clinical settings. According to a 2016 analysis of population data from Scotland, 15% of adults with T1DM had been prescribed metformin at least once, with 8% actively using it at the time [63, 64]. If similar trends are observed globally, it is possible that metformin is being prescribed to thousands of individuals with T1DM worldwide. In France, metformin has had a product licence for use in T1DM since 1996. However, there was a lack of clinical evidence to support this practice [67].

In alignment with earlier research, a meta‐analysis conducted by Liu et al. in adolescents with T1DM indicated that metformin led to a notable reduction in HbA1c level after a year of treatment, as well as favourable changes in BMI after 3 months and BMI Z‐score after 6 months [19]. Additionally, reductions in TIDD were observed at 3, 6, and 12 months. However, despite these positive effects on glycemic control and weight‐related measures, metformin raised HG events and GIAEs risk [19]. In a prior meta‐analysis conducted by Liu et al., eight RCTs were examined, involving a total of 300 participants. The population primarily consisted of patients with T1DM who were insulin‐resistant and required a high daily insulin dosage. The majority of the studies included in the meta‐analysis involved a broader age range, including both adolescents and adults. The findings indicated that metformin use was linked to a decrease in weight, TIDD, and lipid levels, including reductions in TC and LDL. Although GIAEs were more likely to occur, there was no heightened risk of HG or DKA. Additionally, there were no significant changes observed in HbA1c, FPG, or TG levels [58].

Furthermore, in the meta‐analysis by Xu et al., various cardiovascular and metabolic parameters were significantly improved among participants receiving metformin. They found a reduction in carotid intimal media thickness, ascending aortic pulse wave velocity, and descending aortic wall shear stress, indicating potential benefits for vascular health [59]. Additionally, participants experienced a decrease in TIDD and body weight, alongside reductions in fat‐free mass and BMI. The study also noted improvements in metabolic parameters, such as increased flow‐mediated dilation and enhanced glucose infusion rates relative to insulin levels, suggesting improved insulin sensitivity. However, in their study, metformin did not lead to significant changes in waist circumference, reactive hyperemia index, blood pressure, BMI Z‐score, and levels of TC, TG, and HDL [59]. A meta‐analysis performed by Al Khalifah et al., encompassing six randomised trials with 325 adolescents, found that metformin did not significantly impact HbA1c level. However, BMI (MD = −1.46 [95% CI: −2.54, −0.38], *p* < 0.01) and insulin dosage (MD = −0.15 units/kg, [95% CI: 0.24, −0.06], *p* < 0.01) showed marked improvements [16]. Additionally, two other meta‐analyses [11, 15] examined the effect of metformin in T1DM patients, but they did not perform a subgroup analysis specifically for adolescents and adults. In contrast to those earlier analyses, this updated systematic review and meta‐analysis incorporated a greater number of RCTs (29 studies involving 2051 patients), leading to more solid and dependable results. Significantly, we executed a subgroup analysis to independently evaluate the effects of metformin adjunct therapy.

In addition to metformin, newer glucose‐lowering agents like SGLT2 inhibitors and GLP‐1 receptor agonists are being explored as adjunct therapies in T1DM. GLP‐1 receptor agonists have shown modest reductions in HbA1c and improvements in glucose control, but they may carry a higher risk of HG and a longer time below the target glycemic range, which can limit their use in T1DM. These agents also contribute to weight loss, making them appealing for overweight patients [68]. SGLT2 inhibitors, on the other hand, significantly reduce glucose variability and improve kidney function, alongside reductions in HbA1c. They also show cardiovascular benefits, but they carry a higher risk of DKA, particularly in insulin‐treated T1DM patients [69, 70]. In comparison, metformin consistently reduces HbA1c, insulin dosage, and lipid levels in both adolescents and adults with T1DM, while also improving metabolic control. It offers a safer profile with lower risk for HG and DKA compared to GLP‐1RAs and SGLT2 inhibitors. Despite gastrointestinal side effects, metformin remains a valuable option for improving metabolic control in T1DM patients.

## 5 Strengths and Limitations

This study benefits from several strengths, including a large sample size of 29 RCTs involving 2051 participants, which enhances the statistical power and reliability of the findings. Additionally, we conducted subgroup analyses to separately evaluate the effects of metformin in adolescents and adults, providing a more nuanced understanding of its impact across age groups. By incorporating recently published studies, our meta‐analysis offers updated insights into the efficacy and safety of metformin as adjunct therapy in T1DM. However, our study has several limitations: (a) the identified RCTs had small sample sizes, which raise the risk of exaggerating metformin adjunct therapy effectiveness in T1DM patients; (b) variations in factors such as diabetes duration, male patient ratio, metformin dosage, and BMI of patients may also influence the overall results, as we lacked sufficient data to perform subgroup analyses; (c) while T1DM is known to increase the risk of CVD, the effects of metformin on CVD were not addressed in this meta‐analysis; and (d) we were unable to access unpublished data, as no attempts were made to contact study authors for additional information. Since the scope of this review was limited to published data, the findings were based on the available published studies. While unpublished data could provide further insights, future studies may benefit from considering this avenue to further enhance the robustness of the evidence base; (e) finally, due to the significant heterogeneity observed in multiple outcomes (e.g., BMI, HbA1c, TC levels), we did not perform meta‐regression analyses. Although meta‐regression could help identify potential sources of heterogeneity, the complexity of the variations across studies and the limited availability of moderator variables across trials made this analysis challenging. Future studies with more homogeneous data and detailed patient characteristics may benefit from incorporating meta‐regression to better understand the sources of variability in treatment effects.

## 6 Conclusions

Our analyses showed that in T1DM adolescents, metformin adjunct therapy may reduce TIDD at three and nine, as well as levels of HbA1c at three, TC at three, TG at nine, and LDL at 3 months. BMI and TIDD may decrease in T1DM adults using metformin adjunct therapy at 3, 6, and 12 months. Moreover, it may lower levels of HbA1c at 6 and 12 months and TC at 6 months in adults, and raise GIAEs risk in both T1DM adolescents and adults. Nevertheless, more studies are necessary to establish metformin adjunct therapy duration and ideal dosage, as well as to conduct subgroup analyses regarding BMI of patients.

## Author Contributions

**Mohammad Mahdi Masouri:** conceptualisation, methodology, validation, investigation, data curation, writing‐original draft, writing‐review and editing, visualisation, project administration. **Rasoul Ebrahimi:** investigation, software, formal analysis, data curation, writing‐original draft. **Shokoofe Noori:** conceptualisation, methodology, investigation, data curation, writing‐review and editing, supervision.

## Ethics Statement

The study was approved by the Ethical Review Board of the School of Medicine, Shahid Beheshti University of Medical Sciences, Tehran (IR.SBMU.MSP.REC.1403.549).

## Consent

The authors have nothing to report.

## Conflicts of Interest

The authors declare no conflicts of interest.

## Supporting information


Appendix S1.

**Appendix S2.** PRISMA checklist.

## Data Availability

The data sets used and/or analysed during the current study are available from the corresponding author upon reasonable request.
