# Supplementary material for: An Updated Systematic Review and Meta‐Analysis on the Efficacy and Safety of Metformin as Add‐on Therapy to Insulin in Patients With Type 1 Diabetes
Source: Endocrinol Diabetes Metab. 2025 Jun 13;8(4):e70060. doi: 10.1002/edm2.70060 (PMC12165280; doi:10.1002/edm2.70060)
Supplement: Supplementary file 1 — Appendix S1. [file EDM2-8-e70060-s002.docx]

**An updated systematic review and meta-analysis on the efficacy and safety of metformin as add-on therapy to insulin in patients with type 1 diabetes**

**Appendix A. Supplementary material**

**Table of Contents**

**Appendix A.1.** Database search strategies  **3**

**Appendix A.2.** Summary of study designs and baseline data **5**

**Appendix A.3.** Summary of subgroup analysis and Egger's and Begg's tests  **8**

**Appendix A.4.** RoB 2 quality assessment summaries  **12**

**Appendix A.5.** RoB 2 quality assessment graphs  **14**

**Appendix A.6.** Forest plots showing the BMI Z-score of adolescents **15**

**Appendix A.7.** Forest plots showing the TC level of adolescents **16**

**Appendix A.8.** Forest plots showing the TC level of adults  **17**

**Appendix A.9.** Forest plots showing the TG level of adolescents **18**

**Appendix A.10.** Forest plots showing the TG level of adults **19**

**Appendix A.11.** Forest plots showing the LDL level of adolescents **20**

**Appendix A.12.** Forest plots showing the LDL level of adults **21**

**Appendix A.13.** Forest plots showing the HDL level of adolescents **22**

**Appendix A.14.** Forest plots showing the HDL level of adults **23**

**Appendix A.15.** Forest plot showing the DKA risk of adolescents  **24**

**Appendix A.16.** Forest plot showing the DKA risk of adults **25**

**Appendix A.17.** Forest plot showing the HG risk of adolescents **26**

**Appendix A.18.** Forest plot showing the HG risk of adults **27**

**Appendix A.19.** Funnel plots of publication bias for the comparative efficacy **28**

**Appendix A.20.** Funnel plots of publication bias for the comparative safety **30**

**References 31**

**Appendix A.1. Database search strategies.** Search strategy for PubMed, Embase, the Cochrane Central Registry of Controlled Trials (CENTRAL), and Google Scholar.

| Database  (search date) | Search strategy | | Results |
| --- | --- | --- | --- |
|  | Step | Query |  |
| PubMed  (December 14, 2024) | #1 | Diabetes Mellitus, Type 1[MeSH Terms] | 90,149 |
|  | #2 | ((((((((((((((((((Diabetes Mellitus, Type 1[Title/Abstract]) OR (Type 1 Diabetes[Title/Abstract])) OR (Diabetes Mellitus, Insulin Dependent[Title/Abstract])) OR (Insulin-Dependent Diabetes Mellitus[Title/Abstract])) OR (Diabetes Mellitus, Juvenile Onset[Title/Abstract])) OR (Juvenile-Onset Diabetes Mellitus[Title/Abstract])) OR (IDDM[Title/Abstract])) OR (Diabetes Mellitus, Type I[Title/Abstract])) OR (Diabetes Mellitus, Sudden Onset[Title/Abstract])) OR (Sudden-Onset Diabetes Mellitus[Title/Abstract])) OR (Type 1 Diabetes Mellitus[Title/Abstract])) OR (Insulin-Dependent Diabetes Mellitus 1[Title/Abstract])) OR (Insulin Dependent Diabetes Mellitus 1[Title/Abstract])) OR (Juvenile-Onset Diabetes[Title/Abstract])) OR (Juvenile Onset Diabetes[Title/Abstract])) OR (Autoimmune Diabetes[Title/Abstract])) OR (Brittle Diabetes Mellitus[Title/Abstract])) OR (Diabetes Mellitus, Ketosis Prone[Title/Abstract])) OR (Ketosis-Prone Diabetes Mellitus[Title/Abstract]) | 77,644 |
|  | #3 | #1 OR #2 | 117,258 |
|  | #4 | Metformin[MeSH Terms] | 19,353 |
|  | #5 | (((((Metformin[Title/Abstract]) OR (Dimethylbiguanidine[Title/Abstract])) OR (Dimethylguanylguanidine[Title/Abstract])) OR (Glucophage[Title/Abstract])) OR (Metformin Hydrochloride[Title/Abstract])) OR (Metformin HCl[Title/Abstract]) | 31,124 |
|  | #6 | #4 OR #5 | 33,046 |
|  | #7 | random* | 1,830,712 |
|  | #8 | #3 AND #6 AND #7 | 175 |
| Embase  (December 14, 2024) | #1 | 'insulin dependent diabetes mellitus'/exp | 169,795 |
|  | #2 | 'diabetes mellitus, type 1':ti,ab,kw OR 'type 1 diabetes':ti,ab,kw OR 'diabetes mellitus, insulin dependent':ti,ab,kw OR 'insulin-dependent diabetes mellitus':ti,ab,kw OR 'diabetes mellitus, juvenile onset':ti,ab,kw OR 'juvenile-onset diabetes mellitus':ti,ab,kw OR 'iddm':ti,ab,kw OR 'diabetes mellitus, type i':ti,ab,kw OR 'diabetes mellitus, sudden onset':ti,ab,kw OR 'sudden-onset diabetes mellitus':ti,ab,kw OR 'type 1 diabetes mellitus':ti,ab,kw OR 'insulin-dependent diabetes mellitus 1':ti,ab,kw OR 'insulin dependent diabetes mellitus 1':ti,ab,kw OR 'juvenile-onset diabetes':ti,ab,kw OR 'juvenile onset diabetes':ti,ab,kw OR 'autoimmune diabetes':ti,ab,kw OR 'brittle diabetes mellitus':ti,ab,kw OR 'diabetes mellitus, ketosis prone':ti,ab,kw OR 'ketosis-prone diabetes mellitus':ti,ab,kw | 117,127 |
|  | #3 | #1 OR #2 | 195,323 |
|  | #4 | 'metformin'/exp | 96,019 |
|  | #5 | 'metformin':ti,ab,kw OR 'dimethylbiguanidine':ti,ab,kw OR 'dimethylguanylguanidine':ti,ab,kw OR 'glucophage':ti,ab,kw OR 'metformin hydrochloride':ti,ab,kw OR 'metformin hcl':ti,ab,kw | 52,657 |
|  | #6 | #4 OR #5 | 99,803 |
|  | #7 | 'random*' | 2,437,957 |
|  | #8 | #3 AND #6 AND #7 | 1,021 |
| Cochrane  (December 14, 2024) | #1 | MeSH Descriptor: [Diabetes Mellitus, Type 1] explode all trees | 7,765 |
|  | #2 | ("Diabetes Mellitus, Type 1"):ti,ab,kw OR ("Type 1 Diabetes"):ti,ab,kw OR ("Diabetes Mellitus, Insulin Dependent"):ti,ab,kw OR ("Insulin-Dependent Diabetes Mellitus"):ti,ab,kw OR ("Diabetes Mellitus, Juvenile Onset"):ti,ab,kw OR ("Juvenile-Onset Diabetes Mellitus"):ti,ab,kw OR ("IDDM"):ti,ab,kw OR ("Diabetes Mellitus, Type I"):ti,ab,kw OR ("Diabetes Mellitus, Sudden Onset"):ti,ab,kw OR ("Sudden-Onset Diabetes Mellitus"):ti,ab,kw OR ("Type 1 Diabetes Mellitus"):ti,ab,kw OR ("Insulin-Dependent Diabetes Mellitus 1"):ti,ab,kw OR ("Insulin Dependent Diabetes Mellitus 1"):ti,ab,kw OR ("Juvenile-Onset Diabetes"):ti,ab,kw OR ("Juvenile Onset Diabetes"):ti,ab,kw OR ("Autoimmune Diabetes"):ti,ab,kw OR ("Brittle Diabetes Mellitus"):ti,ab,kw OR ("Diabetes Mellitus, Ketosis Prone"):ti,ab,kw OR ("Ketosis-Prone Diabetes Mellitus"):ti,ab,kw | 35,332 |
|  | #3 | #1 OR #2 | 35,333 |
|  | #4 | MeSH Descriptor: [Metformin] explode all trees | 5,549 |
|  | #5 | ("Metformin"):ti,ab,kw OR ("Dimethylbiguanidine"):ti,ab,kw OR ("Dimethylguanylguanidine"):ti,ab,kw OR ("Glucophage"):ti,ab,kw OR ("Metformin Hydrochloride"):ti,ab,kw OR ("Metformin HCl"):ti,ab,kw | 14,205 |
|  | #6 | #4 OR #5 | 14,205 |
|  | #7 | random* | 1,486,447 |
|  | #8 | #3 AND #6 AND #7 | 3,655 |
| Google Scholar  (December 17, 2024) | #1 | "Metformin" AND ("Type 1 diabetes" OR "diabetes") | 757,000 |

**Appendix A.2. Summary of study designs and baseline data.** Study design and baseline characteristics of the included studies.

| First author  Publication year  Trial registration | Country | Study design | Population | Follow-up duration (m) | Intervention, dose (mg/d) | Weight (kg) | | Patients (n) | Male (n) | Lost to follow-up (n) | Age (y) | | T1DM duration (y) | | Efficacy outcomes | Safety outcomes | Other outcomes |
| --- | --- | --- | --- | --- | --- | --- | --- | --- | --- | --- | --- | --- | --- | --- | --- | --- | --- |
|  |  |  |  |  |  | Mean | SD |  |  |  | Mean | SD | Mean | SD |  |  |  |
| Amina, et al.  2023 [1]  NA | Pakistan | P, OL, SC | Patients aged ≥ 12 y with T1DM | 3 | Metformin, 250-2,000 | NA | | 30 | 17 | 0 | 20.8 | 6.1 | 8.7 | 6.3 | BMI and HbA1c | NA | A significant difference was observed in RBG.  No significant difference was observed in FPG. |
|  |  |  |  |  | Placebo | NA | | 50 | 31 | 0 | 21.7 | 5.5 | 10.1 | 5.8 |  |  |  |
| Anderson, et al.  2017 [2]  ACTRN12611000148976 | Australia | P, DB, SC | Patients aged 8-18 y with T1DM at least 0.5 y in duration, BMI ≥ 50^th^ percentile, and insulin dosage > 0.5 unit/kg/d | 12 | Metformin, 2,000 | NA | | 45 | 21 | 1 | 14.0 | 2.5 | 5.2 | 3.6 | TIDD, HbA1c, TC, TG, LDL, and HDL | DKA, GIAEs, and HG | Significant differences were observed in leptin, DXA lean mass, and vitamin B12, and GTN improved.  No significant differences were observed in carotid/aortic IMT, BP, waist and hip circumference, fat mass, hsCRP, adiponectin, adiponectin/leptin ratio, UACR, FMD, DXA fat, DXA fat mass, homocysteine, lactate, AST, ALT, and serum creatinine. |
|  |  |  |  |  | Placebo | NA | | 45 | 20 | 0 | 13.3 | 2.6 | 5.8 | 4.1 |  |  |  |
| Bjornstad, et al.  2018 [3]  NCT01808690 | USA | P, DB, SC | Patients aged 12-21 y with T1DM at least 1 y in duration, HbA1c ≤ 12 %, and BMI ≥ 5^th^ percentile | 3 | Metformin, 2,000 | 72.3 | 13.7 | 25 | 13 | 1 | 17.3 | 2.3 | 7.8 | 4.4 | BMI, BMI Z-score, TIDD, HbA1c, TC, TG, LDL, and HDL | DKA, GIAEs, and HG | Significant differences were observed in glucose infusion rate/insulin, body weight, fat mass, AA WSS_MAX_, AA PWV, and far-wall diastolic carotid IMT.  No significant differences were observed in SBP, DBP, AA WSS_TA_, DA WSS_MAX_, DA WSS_TA_, DA PWV, brachial distensibility, hemoglobin, AST, ALT, and serum creatinine. |
|  |  |  |  |  | Placebo | 69.9 | 14.6 | 23 | 11 | 2 | 15.9 | 2.7 | 9.3 | 5.1 |  |  |  |
| Burchardt, et al.  2016 [4]  NCT01889706 | Poland | P, OL, SC | Patients aged 18-60 y with T1DM at least 5 y in duration and HbA1c > 7.5 % | 6 | Metformin, 500-1,500 | 90.0 | 15.0 | 42 | 19 | 0 | 35.2 | 11.0 | NA | | HbA1c, TG, LDL, and HDL | DKA, GIAEs, and HG | Significant differences were observed in body weight, IMT, CEL, FPG, and mean glycemia concentrations. |
|  |  |  |  |  | Placebo | 80.9 | 9.7 | 42 | 29 | 0 | 31.2 | 11.7 | NA | |  |  |  |
| Burchardt, et al.  2013 [5]  NA | Poland | P, OL, SC | Patients aged 18-60 y with T1DM at least 5 y in duration and HbA1c > 7.5 % | 6 | Metformin, 1,000–2,550 | NA | | 35 | 35 | 2 | 35.3 | 11.2 | 15.9 | 7.8 | BMI, HbA1c, TC, TG, LDL, and HDL | NA | Significant differences were observed in ADPG, FPG, and PPG. |
|  |  |  |  |  | Placebo | NA | | 33 |  | 14 | 30.5 | 10.6 | 15.8 | 7.7 |  |  |  |
| Codner, et al.  2013 [6]  ISRCTN51650949 | Chile | P, DB, SC | Patients aged < 21 y with T1DM at least 1.5 y in duration, HbA1c ≥ 7.0 %, and insulin dosage ≥ 0.5 unit/kg/d | 9 | Metformin, 1,700 | NA | | 13 | NA | 0 | 17.7 | 1.6 | 9.3 | 5.1 | BMI, HbA1c, and TIDD | DKA, GIAEs, and HG | Significant differences were observed in estradiol, 17-OH progesterone, testosterone, free androgen index, and androstenedione.  No significant differences were observed in Ferriman-Gallwey scores, menstrual cycle duration, ovulatory rate, LH, FSH, SHBG, and DHEAS. |
|  |  |  |  |  | Placebo | NA | | 11 |  | 1 | 16.7 | 1.7 | 5.5 | 3.1 |  |  |  |
| Cree-Green, et al.  2019 [7]  NCT02045290 | USA | P, DB, MC | Patients aged 12-<20 y with T1DM, 7.5 % ≤ HbA1c ≤ 9.9 %, BMI ≥ 85^th^ percentile, and insulin dosage ≥ 0.8 unit/kg/d | 3 | Metformin, 500-1,000 | 86.7 | 17.0 | 19 | 7 | 0 | 15.8 | 2.1 | NA | | BMI Z-score, TIDD, and HbA1c | DKA, GIAEs, and HG | Significant differences were observed in body weight and glucose infusion rate.  No significant differences were observed in FFM and waist circumference. |
|  |  |  |  |  | Placebo | 85.2 | 17.7 | 18 | 10 | 1 | 15.5 | 2.2 | NA | |  |  |  |
| Elbarbary, et al.  2022 [8]  NCT04879511 | Egypt | P, DB, SC | Patients aged 12-18 y with T1DM at least 5 y in duration | 6 | Metformin, 500 | NA | | 40 | 24 | 2 | 15.6 | 1.6 | 10.2 | 3.7 | BMI Z-score, TIDD, HbA1c, TC, and TG | DKA, GIAEs, and HG | Significant differences were observed in FPG, RBG, SBP, DBP, hsCRP, UACR, carotid IMT, and Nrg-4.  No significant differences were observed in serum creatinine and eGFR. |
|  |  |  |  |  | Placebo | NA | | 40 | 22 | 3 | 15.3 | 1.7 | 9.0 | 2.3 |  |  |  |
| Gourgari, et al.  2021 [9]  NA | USA | P, DB, MC | Patients aged 12-20 y with T1DM, 7.5 % ≤ HbA1c ≤ 10.0 %, BMI ≥ 85^th^ percentile, and insulin dosage ≥ 0.8 unit/kg/d | 6 | Metformin, 500-2,000 | NA | | 25 | 6 | 0 | 15.6 | 1.6 | NA | | BMI, BMI Z-score, TIDD, HbA1c, TC, TG, LDL, and HDL | NA | Significant differences were observed in PGRP2 and A2MG.  No significant differences were observed in CEC, VLDL, DXA fat, DXA fat mass, and waist circumference. |
|  |  |  |  |  | Placebo | NA | | 10 | 3 | 0 | 15.5 | 1.7 | NA | |  |  |  |
| Hamilton, et al.  2003 [10]  NA | Canada | P, DB, SC | Patients aged 12-17 y with T1DM at least 3 y in duration, 8.0 % < HbA1c < 11.0 %, and insulin dosage ≥ 1.0 unit/kg/d | 3 | Metformin, 500-2,000 | 62.9 | 13.7 | 14 | 6 | 1 | 15.9 | 1.9 | 9.9 | 4.4 | BMI, TIDD, and HbA1c | DKA, GIAEs, and HG | A significant difference was observed in FPG.  No significant difference was observed in insulin sensitivity. |
|  |  |  |  |  | Placebo | 63.3 | 13.6 | 13 | 7 | 2 | 16.0 | 1.7 | 7.0 | 3.8 |  |  |  |
| Jacobsen, et al.  2009 [11]  NA | Denmark | P, DB, SC | Patients aged 18-60 y with T1DM at least 1 y in duration, HbA1c ≥ 8.0 %, and BMI ≥ 25.0 kg/m^2^ | 6 | Metformin, 2,000 | 87.6 | 13.2 | 12 | 14 | 0 | 43.5 | 13.1 | 17.8 | 10.3 | TIDD, HbA1c, TC, TG, LDL, and HDL | DKA and GIAEs | A significant difference was observed in body weight.  No significant differences were observed in SBP, DBP, FPG, and PPG. |
|  |  |  |  |  | Placebo | 92.0 | 10.2 | 12 |  | 1 | 37.3 | 9.6 | 20.3 | 10.2 |  |  |  |
| Janić, et al.  2024 [12]  NCT03639545 | Slovenia | P, DB, SC | Patients aged 30-65 y with T1DM and BMI ≥ 25.0 kg/m^2^ | 3 | Metformin, 2,000 | 86.9 | 1.8 | 10 | NA | 0 | 46.4 | 3.9 | 23.1 | 4.8 | NA | DKA and HG | No significant differences were observed in serum creatinine, eGFR, eDP, UACR, uric acid, AST, ALT, γ-GT, FIB-4, FLI, and NAFLD fibrosis score. |
|  |  |  |  |  | Placebo | 88.1 | 2.0 | 10 |  | 0 | 43.1 | 2.1 | 22.2 | 3.8 |  |  |  |
| Khan, et al.  2006 [13]  NA | UK | Cr, DB, SC | Patients with T1DM at least 1 y in duration, HbA1c > 6.1 %, and BMI > 27.0 kg/m^2^ | 4 | Metformin, 2,550 | 91.0 | 12.0 | 8 | 8 | 3 | NA | | NA | | HbA1c, TC, TG, LDL, and HDL | DKA, GIAEs, and HG | A significant difference was observed in FPG.  No significant difference was observed in body weight. |
|  |  |  |  |  | Placebo | 91.0 | 12.0 | 7 |  |  | NA | | NA | |  |  |  |
| Libman, et al.  2015 [14]  NCT01881828 | USA | P, DB, MC | Patients aged 12-<20 y with T1DM, 7.5 % ≤ HbA1c ≤ 9.9 %, BMI ≥ 85^th^ percentile, and insulin dosage ≥ 0.8 unit/kg/d | 6 | Metformin, 500-2,000 | 77.0 | 1.3 | 71 | 27 | 1 | 15.4 | 1.7 | 7.5 | 3.6 | BMI Z-score, TIDD, HbA1c, TC, TG, LDL, and HDL | DKA, GIAEs, and HG | Significant differences were observed in body weight, DXA fat mass, and DXA fat.  No significant differences were observed in waist circumference, DXA lean, SBP percentile, DBP percentile, and VLDL. |
|  |  |  |  |  | Placebo | 76.3 | 1.1 | 69 | 21 | 0 | 15.1 | 1.8 | 6.4 | 3.0 |  |  |  |
| Lund, et al.  2008 [15]  NCT00118937 | Denmark | P, DB, SC | Patients aged ≥ 18 y with T1DM at least 5 y in duration and HbA1c ≥ 8.5 % | 12 | Metformin, 2,000 | 80.5 | 12.5 | 49 | 33 | 2 | 46.1 | 11.6 | 30.0 | NA | BMI, TIDD, and HbA1c | DKA, GIAEs, and HG | Significant differences were observed in body weight and hip circumference.  No significant differences were observed in waist circumference, waist/hip ratio, and FPG. |
|  |  |  |  |  | Placebo | 79.0 | 15.3 | 51 | 31 | 6 | 44.9 | 10.8 | 26.0 | NA |  |  |  |
| Lund, et al.  2009 [16]  NA | Denmark | P, DB, SC | Patients aged 10-20 y with T1DM and HbA1c ≥ 8.5 % | 12 | Metformin, 2,000 | 80.5 | 12.5 | 49 | 33 | 2 | 46.1 | 11.6 | 30.0 | NA | TIDD, TC, TG, LDL, and HDL | NA | NA |
|  |  |  |  |  | Placebo | 79.0 | 15.3 | 51 | 31 | 6 | 44.9 | 10.8 | 26.0 | NA |  |  |  |
| Lunder, et al.  2018 [17]  NCT03639545 | Slovenia | P, DB, SC | Patients aged 30-65 y with T1DM | 3 | Metformin, 2,000 | NA | | 10 | 10 | 0 | 46.4 | 3.9 | 23.2 | 4.8 | BMI and HbA1c | NA | Significant differences were observed in brachial artery FMD and RHI. |
|  |  |  |  |  | Placebo | NA | | 10 | 10 | 0 | 43.1 | 2.1 | 22.2 | 3.8 |  |  |  |
| Meyer, et al.  2002 [18]  NA | France | P, DB, SC | Patients with T1DM and HbA1c < 9 % | 6 | Metformin, 1,700 | 78.4 | 18.1 | 31 | 17 | 0 | 39.9 | 12.9 | 16.9 | 8.9 | TIDD, HbA1c, TC, and TG | DKA, GIAEs, and HG | NA |
|  |  |  |  |  | Placebo | 74.5 | 11.7 | 31 | 20 | 0 | 41.1 | 9.8 | 21.6 | 10.2 |  |  |  |
| Mondkar, et al.  2024 [19]  CTRI/2019/11/022126 | India | P, DB, SC | Patients aged 10-19 y with T1DM at least 1 y in duration and HbA1c > 8.0 % | 9 | Metformin, 1,000-2,000 | NA | | 41 | 21 | 1 | 14.8 | 3.1 | 5.3 | 2.2 | BMI Z-score, TIDD, HbA1c, TC, TG, LDL, and HDL | DKA, GIAEs, and HG | A significant difference was observed in waist circumference Z-score.  No significant differences were observed in carotid IMT, weight Z-score, and fat Z-score. |
|  |  |  |  |  | Placebo | NA | | 41 | 21 | 2 | 14.7 | 2.8 | 5.1 | 2.2 |  |  |  |
| Nadeau, et al.  2015 [20]  NA | USA | P, DB, SC | Patients aged 13-20 y with T1DM at least 1 y in duration and HbA1c > 8.5 % | 6 | Metformin, 1,000-2,000 | 65.7 | 12.3 | 40 | 47 | 12 | 15.9 | 1.7 | 6.7 | 3.6 | BMI, BMI Z-score, TIDD, HbA1c, TC, TG, LDL, and HDL | DKA, GIAEs, and HG | No significant differences were observed in SBP, DBP, waist circumference, and body weight. |
|  |  |  |  |  | Placebo | 67.1 | 13.2 | 40 |  | 9 | 16.0 | 1.6 | 6.3 | 3.5 |  |  |  |
| Nwosu, et al.  2015 [21]  NCT01334125 | USA | P, DB, SC | Patients aged 10-18 y with T1DM at least 1 y in duration, 8.0 % < HbA1c < 14 %, and BMI > 85^th^ percentile | 9 | Metformin, 1,000 | 75.5 | 25.0 | 15 | 8 | 3 | 15.0 | 2.5 | 5.7 | 4.4 | BMI, TIDD, and HbA1c | DKA, GIAEs, and HG | No significant differences were observed in FPG, ICR, ISF, IBG, waist circumference, and body weight. |
|  |  |  |  |  | Placebo | 70.8 | 17.9 | 13 | 5 | 3 | 14.5 | 3.1 | 5.7 | 5.0 |  |  |  |
| Oza, et al.  2023 [22]  CTRI/2019/11/022126 | India | P, DB, SC | Patients aged 10-19 y with T1DM at least 1 y in duration | 3 | Metformin, 1,000-2,000 | NA | | 32 | 32 | 0 | 13.8 | 2.5 | 5.0 | 2.2 | BMI Z-score, TIDD, HbA1c, TC, TG, LDL, and HDL | DKA, GIAEs, and HG | No significant differences were observed in SBP, DBP, weight Z-score, waist circumference, hip circumference, waist Z-score, waist/hip ratio, lactate, VLDL, AST, ALT, fat percentage Z-score, LBM percentage Z-score, and eGDR. |
|  |  |  |  |  | Placebo | NA | | 27 |  | 0 | 13.8 | 2.4 | 5.2 | 2.5 |  |  |  |
| Petrie, et al.  2017 [23]  NCT01483560 | Australia  Canada  Denmark  the Netherland  UK | P, DB, MC | Patients aged ≥ 40 y with T1DM at least 5 y in duration, HbA1c > 8.0 %, and BMI ≥ 27 kg/m^2^ | 36 | Metformin, 2,000 | 83.9 | 15.4 | 219 | 129 | 26 | 55.2 | 8.5 | 33.4 | 11.0 | TIDD, HbA1c, and LDL | DKA and GIAEs | Significant differences were observed in maximal carotid far-wall IMT, body weight, and eGFR.  No significant differences were observed in mean carotid far-wall IMT and RHI. |
|  |  |  |  |  | Placebo | 83.5 | 13.7 | 209 | 124 | 15 | 55.8 | 8.8 | 34.3 | 10.5 |  |  |  |
| Pitocco, et al.  2013 [24]  NA | Italy | P, DB, MC | Patients aged ≥ 18 y with T1DM at least 5 y in duration and HbA1c ≥ 10.0 % | 6 | Metformin, 850-2,550 | 83.0 | 12.0 | 21 | 9 | 0 | 46.0 | 8.0 | 9.2 | 0.7 | BMI | DKA, GIAEs, and HG | Significant differences were observed in body weight, FMD, and PGF2α.  No significant differences were observed in NMD and GV. |
|  |  |  |  |  | Placebo | 77.0 | 11.0 | 21 | 9 | 0 | 41.0 | 10.0 | 8.8 | 0.8 |  |  |  |
| Särnblad, et al.  2003 [25]  NA | Sweden | P, DB, MC | Patients aged 14-20 y with T1DM, HbA1c > 8.0 %, and insulin dosage > 0.9 unit/kg/d | 3 | Metformin, 500-2,000 | 70.9 | 6.8 | 16 | 5 | 5 | 17.2 | 1.7 | 9.1 | 5.0 | BMI, TIDD, and HbA1c | DKA, GIAEs, and HG | No significant differences were observed in body weight, waist circumference, waist/hip ratio, IGF-I, and IGFBP-1. |
|  |  |  |  |  | Placebo | 69.0 | 10.7 | 14 | 4 | 1 | 16.9 | 1.4 | 7.1 | 3.0 |  |  |  |
| Sheikhy, et al.  2022 [26]  IRCT20201207049638N1 | Iran | P, DB, SC | Patients aged 10-20 y with T1DM at least 1 y in duration, HbA1c > 8.0 %, and insulin dosage > 1.0 unit/kg/d | 9 | Metformin, 1,000-2,000 | NA | | 26 | 10 | 1 | 12.5 | 1.3 | 5.3 | 1.0 | TIDD, HbA1c, TC, TG, LDL, and HDL | DKA and HG | A significant difference was observed in serum creatinine.  No significant differences were observed in AST, ALT, and BUN. |
|  |  |  |  |  | Placebo | NA | | 26 | 9 | 1 | 13.0 | 2.1 | 4.8 | 1.5 |  |  |  |
| Yang, et al.  2020 [27]  NA | China | Cr, OL, SC | Patients aged 12-18 y with T1DM at least 1 y in duration, 7.5 % ≤ HbA1c ≤ 10 %, and insulin dosage ≥ 0.8 unit/kg/d | 6 | Metformin, 500-1,000 | NA | | 9 | 6 | 1 | 15.2 | 2.3 | 4.7 | 2.2 | BMI, TIDD, HbA1c, TC, TG, LDL, and HDL | DKA, GIAEs, and HG | No significant differences were observed in FPG, SBP, DBP, MBG, MAGE, and MODD. |
|  |  |  |  |  | Placebo | NA | | 8 | 4 | 2 | 13.4 | 1.8 | 4.0 | 1.0 |  |  |  |
| Zawada, et al.  2018 [28]  NCT01889706 | Poland | P, OL, SC | Patients aged > 18 y with T1DM at least 3 y in duration and HbA1c > 7.5 % | 6 | Metformin, 500–2,550 | NA | | 74 | 39 | 0 | 32.0 | 26.3 | 13.0 | 28.5 | BMI, TIDD, HbA1c, TC, TG, LDL, and HDL | DKA, GIAEs, and HG | Significant differences were observed in waist circumference, SBP, DBP, TBF, trunk body fat, abdominal visceral body fat, eGDR, FPG, PPG, MPG, and eGFR.  No significant differences were observed in AST and ALT. |
|  |  |  |  |  | Placebo | NA | | 40 | 15 | 0 | 27.5 | 25.0 | 15.0 | 11.7 |  |  |  |
| Zhang, et al.  2021 [29]  NCT03590262 | China | P, OL, SC | Patients aged 18-75 y with T1DM | 3 | Metformin, 1,000-2,000 | 62.6 | 7.6 | 34 | 14 | 2 | 31.0 | 10.0 | 7.3 | 7.8 | BMI, TIDD, HbA1c, TC, TG, LDL, and HDL | DKA, GIAEs, and HG | Significant differences were observed in MAGE, body weight, SDBG, LAGE, and MSG.  No significant differences were observed in MODD and TIR. |
|  |  |  |  |  | Placebo | 62.2 | 7.6 | 31 | 12 | 1 | 32.0 | 11.0 | 10.7 | 13.2 |  |  |  |

**Abbreviations:** **AA**, ascending aorta; **ADPG**, average daily plasma glucose; **ALT**, alanine aminotransferase; **A2MG**, alpha-2-macroglobulin; **AST**, aspartate aminotransferase; **BMI**, body mass index; **BP**, blood pressure; **BUN**, blood urea nitrogen; **CEC**, cholesterol efflux capacity; **CEL**, cholesteryl ester lipase; **Cr**, crossover; **DA**, descending aorta; **DB**, double-blind; **DBP**, diastolic blood pressure; **DHEAS**, dehydroepiandrosterone sulfate; **DKA**, diabetic ketoacidosis; **DXA**, dual-energy x-ray absorptiometry; **eDP**, estimated daily proteinuria; **eGDR**, estimated glucose disposal rate; **eGFR**, estimated glomerular filtration rate; **FFM**, fat-free mass; **FIB-4**, fibrosis-4 index; **FLI**, fatty liver index; **FMD**, flow mediated dilatation; **FPG**, fasting plasma glucose; **FSH**, follicle-stimulating hormone; **γ-GT**, gamma-glutamyl transferase; **GIAEs**, gastrointestinal adverse events; **GTN**, glyceryl trinitrate mediated dilatation; **GV**, glycemic variability; **HbA1c**, glycosylated hemoglobin; **HDL**, high-density lipoprotein; **HG**, hypoglycemia; **hsCRP**, high sensitivity C-reactive protein; **IBG**, ideal blood glucose; **ICR**, insulin-to-carbohydrate ratio; **IGFBP-1**, IGF-binding protein-1; **IGF-I**, insulin-like growth factor I; **IMT**, intima media thickness; **ISF**, insulin sensitivity factor; **kg**, kilogram; **LAGE**, largest amplitude of glycemic excursions; **LBM**, lean body mass; **LDL**, low-density lipoprotein; **LH**, luteinizing hormone; **m**, month; **MAGE**, mean amplitude glycemic excursions; **MBG**, mean blood glucose; **MC**, multi-center; **mg/d**, milligram/day; **MODD**, means of daily differences; **MPG**, mean plasma glucose; **MSG**, mean sensor glucose; **n**, number; **NA**, not applicable; **NAFLD**, non-alcoholic fatty liver disease; **NMD**, nitrate-mediated dilation; **Nrg-4**, neuregulin-4; **OL**, open-label; **P**, parallel; **PGF2α**, 8-iso-prostaglandin F2α; **PGRP2**, peptidoglycan recognition protein 2; **PPG**, postprandial plasma glucose; **PWV**, pulse wave velocity; **RBG**, Random blood glucose; **RHI**, reactive hyperemia index; **SBP**, systolic blood pressure; **SC**, single-center; **SD**, standard deviation; **SDBG**, standard deviation of blood glucose; **SHBG**, sex hormone binding globulin; **TBF**, total body fat; **TC**, total cholesterol; **T1DM**, type 1 diabetes mellitus; **TG**, triglyceride; **TIDD**, total insulin daily dose; **TIR**, time in range; **UACR**, urine albumin creatinine ratio; **UK**, United Kingdom; **unit/kg/d**, unit/kilogram/day; **USA**, United States of America; **VLDL**, very-low-density lipoprotein; **WSS_MAX_**, maximal wall shear stress; **WSS_TA_**, time-averaged wall shear stress; **y**, year.

**Appendix A.3. Summary of subgroup analysis and Egger's and Begg's tests.** Mean difference and risk ratio subgroup analysis based on age group (adolescent or adult) and follow-up duration for efficacy and safety outcomes, respectively. Egger's and Begg's tests results are also shown.

| Age group | Follow-up duration (m) | Outcome | MD [95%CI] / RR [95%CI] | No of studies | P-value | I^2^ (%) |
| --- | --- | --- | --- | --- | --- | --- |
| Efficacy outcomes | | | | | | |
| Adolescent | 3 | BMI | -0.73 [-1.93, 0.47] | 6 | 0.23 | 95.51 |
|  |  | BMI Z-score | 0.41 [-0.72, 1.55] | 6 | 0.48 | 96.94 |
|  |  | TIDD | -0.61 [-1.02, -0.20] | 10 | 0.00 | 83.47 |
|  |  | HbA1c | -0.45 [-0.79, -0.11] | 12 | 0.01 | 80.02 |
|  |  | TC | -0.78 [-1.54, -0.02] | 5 | 0.04 | 92.46 |
|  |  | TG | -0.27 [-0.68, 0.14] | 5 | 0.20 | 76.54 |
|  |  | LDL | -0.69 [-1.36, -0.02] | 5 | 0.04 | 90.69 |
|  |  | HDL | 0.18 [-0.24, 0.61] | 4 | 0.40 | 68.46 |
|  | 6 | BMI | -0.22 [-0.52, 0.07] | 4 | 0.14 | 0.00 |
|  |  | BMI Z-score | -0.31 [-0.70, 0.08] | 4 | 0.12 | 64.94 |
|  |  | TIDD | -0.79 [-1.83, 0.25] | 7 | 0.13 | 96.45 |
|  |  | HbA1c | -0.45 [-0.94, 0.05] | 8 | 0.08 | 86.77 |
|  |  | TC | -0.47 [-1.14, 0.21] | 6 | 0.18 | 91.29 |
|  |  | TG | -0.03 [-0.45, 0.40] | 5 | 0.90 | 73.53 |
|  |  | LDL | -0.55 [-1.98, 0.88] | 4 | 0.45 | 96.41 |
|  |  | HDL | -0.16 [-0.62, 0.31] | 5 | 0.51 | 77.96 |
|  | 9 | BMI | -0.07 [-0.60, 0.46] | 2 | 0.80 | 0.00 |
|  |  | BMI Z-score | -0.35 [-0.78, 0.08] | 1 | 0.11 | NA |
|  |  | TIDD | -0.36 [-0.68, -0.03] | 4 | 0.03 | 18.30 |
|  |  | HbA1c | -0.36 [-0.74, 0.03] | 4 | 0.07 | 38.64 |
|  |  | TC | -0.67, [-1.37, 0.02] | 2 | 0.06 | 72.88 |
|  |  | TG | -0.47 [-0.81, -0.13] | 2 | 0.01 | 0.00 |
|  |  | LDL | 0.06 [-0.56, 0.68] | 2 | 0.85 | 68.63 |
|  |  | HDL | -0.14 [-0.52, 0.24] | 2 | 0.47 | 17.50 |
|  | 12 | TIDD | 3.18 [-8.57, 2.21] | 2 | 0.25 | 99.08 |
|  |  | HbA1c | -0.17 [-0.58, 0.24] | 1 | 0.42 | NA |
|  |  | TC | -1.63 [-4.49, 1.22] | 2 | 0.26 | 98.38 |
|  |  | TG | 0.01 [-0.28, 0.30] | 2 | 0.94 | 0.00 |
|  |  | LDL | -1.74 [-4.73, 1.24] | 2 | 0.25 | 98.45 |
|  |  | HDL | -0.74 [-2.20, 0.72] | 2 | 0.32 | 95.58 |
|  | Overall | BMI | -0.44 [-1.01, 0.12] | 12 | 0.12 | 89.53 |
|  |  | **Egger:** 0.0274; **Begg:** 0.0467 | | | | |
|  |  | BMI z-score | 0.06 [-0.55, 0.68] | 11 | 0.84 | 94.83 |
|  |  | **Egger:** 0.0168; **Begg:** 0.3889 | | | | |
|  |  | TIDD | -0.82 [-1.37, -0.26] | 23 | 0.00 | 96.01 |
|  |  | **Egger:** 0.0694; **Begg:** 0.6526 | | | | |
|  |  | HbA1c | -0.42 [-0.65, -0.19] | 25 | 0.00 | 79.33 |
|  |  | **Egger:** 0.5549; **Begg:** 0.5283 | | | | |
|  |  | TC | -0.75 [-1.23, -0.27] | 15 | 0.00 | 93.51 |
|  |  | **Egger:** 0.9411; **Begg:** 1.0000 | | | | |
|  |  | TG | -0.18 [-0.38, 0.03] | 14 | 0.09 | 63.60 |
|  |  | **Egger:** 0.3456; **Begg:** 0.7007 | | | | |
|  |  | LDL | -0.69 [-1.33, -0.05] | 13 | 0.04 | 95.59 |
|  |  | **Egger:** 0.6908; **Begg:** 1.0000 | | | | |
|  |  | HDL | -0.13 [-0.45, 0.18] | 13 | 0.42 | 83.04 |
|  |  | **Egger:** 0.0391; **Begg:** 0.0500 | | | | |
| Adult | 3 | BMI | -1.56 [-2.05, -1.08] | 2 | 0.00 | 0.00 |
|  |  | TIDD | -1.26 [-1.80, -0.72] | 1 | 0.00 | NA |
|  |  | HbA1c | -0.26 [-0.65, 0.13] | 3 | 0.20 | 0.00 |
|  |  | TC | -0.09 [-0.53, 0.34] | 2 | 0.68 | 0.00 |
|  |  | TG | -0.18 [-0.62, 0.26] | 2 | 0.42 | 0.00 |
|  |  | LDL | -0.21 [-0.65, 0.22] | 2 | 0.34 | 0.00 |
|  |  | HDL | 0.14 [-0.29, 0.58] | 2 | 0.52 | 0.00 |
|  | 6 | BMI | -0.71 [-1.23, -0.19] | 3 | 0.01 | 66.28 |
|  |  | TIDD | -0.44 [-0.73, -0.16] | 3 | 0.00 | 0.00 |
|  |  | HbA1c | -0.70 [-1.10, -0.30] | 5 | 0.00 | 66.02 |
|  |  | TC | -0.60 [-1.09, -0.10] | 4 | 0.02 | 69.20 |
|  |  | TG | 0.19 [-0.36, 0.73] | 5 | 0.50 | 82.53 |
|  |  | LDL | -0.14 [-0.38, 0.10] | 4 | 0.25 | 0.00 |
|  |  | HDL | -0.09 [-0.63, 0.44] | 4 | 0.73 | 76.55 |
|  | 12 | BMI | -3.09 [-3.67, -2.51] | 1 | 0.00 | NA |
|  |  | TIDD | -0.73 [-1.13, -0.32] | 1 | 0.00 | NA |
|  |  | HbA1c | 1.17 [0.75, 1.59] | 1 | 0.00 | NA |
|  | 36 | TIDD | 0.00 [-0.19, 0.19] | 1 | 1.00 | NA |
|  |  | HbA1c | -0.12 [-0.31, 0.07] | 1 | 0.23 | NA |
|  |  | LDL | -0.16 [-0.35, 0.03] | 1 | 0.10 | NA |
|  | Overall | BMI | -140 [-2.20, -0.59] | 6 | 0.00 | 91.45 |
|  |  | **Egger:** 0.6546; **Begg:** 0.7071 | | | | |
|  |  | TIDD | -0.57 [-0.97, -0.18] | 6 | 0.00 | 81.79 |
|  |  | **Egger:** 0.0013; **Begg:** 0.1329 | | | | |
|  |  | HbA1c | -0.33 [-0.76, 0.10] | 10 | 0.14 | 88.48 |
|  |  | **Egger:** 0.3245; **Begg:** 0.4743 | | | | |
|  |  | TC | -0.45 [-0.84, -0.07] | 6 | 0.02 | 62.79 |
|  |  | **Egger:** 0.9918; **Begg:** 1.0000 | | | | |
|  |  | TG | 0.06 [-0.30, 0.43] | 7 | 0.73 | 68.64 |
|  |  | **Egger:** 0.0069; **Begg:** 0.0715 | | | | |
|  |  | LDL | -0.16 [-0.30, -0.02] | 7 | 0.03 | 0.00 |
|  |  | **Egger:** 0.4079; **Begg:** 0.2296 | | | | |
|  |  | HDL | 0.03 [-0.25, 0.30] | 6 | 0.85 | 34.58 |
|  |  | **Egger:** 0.0549; **Begg:** 0.4524 | | | | |
| Safety outcomes | | | | | | |
| Adolescent |  | DKA | 1.28 [0.57, 2.86] | 15 | 0.55 | 0.00 |
|  |  | **Egger:** 0.8046; **Begg:** 0.8423 | | | | |
|  |  | GIAEs | 1.74 [1.38, 2.21] | 14 | 0.00 | 0.00 |
|  |  | **Egger:** 0.8347; **Begg:** 0.5112 | | | | |
|  |  | HG | 1.63 [0.73, 3.68] | 15 | 0.24 | 0.00 |
|  |  | **Egger:** 0.4265; **Begg:** 0.1638 | | | | |
| Adult |  | DKA | 0.59 [0.17, 1.98] | 9 | 0.39 | 0.00 |
|  |  | **Egger:** 0.2422; **Begg:** 0.4655 | | | | |
|  |  | GIAEs | 1.31 [1.10, 1.56] | 8 | 0.00 | 70.31 |
|  |  | **Egger:** 0.1206; **Begg:** 0.3865 | | | | |
|  |  | HG | 1.59 [1.01, 2.50] | 7 | 0.05 | 44.88 |
|  |  | **Egger:** 0.0853; **Begg:** 0.7639 | | | | |

**Abbreviations:** **BMI**, body mass index; **DKA**, diabetic ketoacidosis; **GIAEs**, gastrointestinal adverse events; **HbA1c**, glycosylated hemoglobin; **HDL**, high-density lipoprotein; **HG**, hypoglycemia; **LDL**, low-density lipoprotein; **m**, month; **MD**, mean difference; **NA**, not applicable; **RR**, risk ratio; **TC**, total cholesterol; **TG**, triglyceride; **TIDD**, total insulin daily dose.

**Appendix A.4. RoB 2 quality assessment summaries.** RoB 2 summaries for intention-to-treat and per protocol parallel and crossover randomized controlled trials. D_1_, Randomization process; D_S_, Bias arising from period and carryover effects*; D_2_, Deviations from the intended interventions; D_3_, Missing outcome data; D_4_, Measurement of the outcome; D_5_, Selection of the reported result.

**Just for crossover randomized controlled trials.*

**Key:**

| Low risk |  |
| --- | --- |
| Some concerns |  |
| High risk |  |

|  | **D_1_** | **D_S_** | **D_2_** | **D_3_** | **D_4_** | **D_5_** | **Overall** |
| --- | --- | --- | --- | --- | --- | --- | --- |
| **First author, Publication year** | **Intention-to-treat parallel randomized controlled trials** | | | | | | |
| Anderson, et al., 2017 |  |  |  |  |  |  |  |
| Codner, et al., 2013 |  |  |  |  |  |  |  |
| Cree-Green, et al., 2019 |  |  |  |  |  |  |  |
| Elbarbary, et al., 2022 |  |  |  |  |  |  |  |
| Libman, et al., 2015 |  |  |  |  |  |  |  |
| Lund, et al., 2008 |  |  |  |  |  |  |  |
| Meyer, et al., 2002 |  |  |  |  |  |  |  |
| Mondkar, et al., 2024 |  |  |  |  |  |  |  |
| Nadeau, et al., 2015 |  |  |  |  |  |  |  |
| Nwosu, et al., 2015 |  |  |  |  |  |  |  |
| Petrie, et al., 2017 |  |  |  |  |  |  |  |
| Särnblad, et al., 2003 |  |  |  |  |  |  |  |
| Zawada, et al., 2018 |  |  |  |  |  |  |  |
|  | **Per protocol parallel randomized controlled trials** | | | | | | |
| Amina, et al., 2023 |  |  |  |  |  |  |  |
| Bjornstad, et al., 2018 |  |  |  |  |  |  |  |
| Burchardt, et al., 2016 |  |  |  |  |  |  |  |
| Burchardt, et al., 2013 |  |  |  |  |  |  |  |
| Gourgari, et al., 2021 |  |  |  |  |  |  |  |
| Hamilton, et al., 2003 |  |  |  |  |  |  |  |
| Jacobsen, et al., 2009 |  |  |  |  |  |  |  |
| Janić, et al., 2024 |  |  |  |  |  |  |  |
| Lund, et al., 2009 |  |  |  |  |  |  |  |
| Lunder, et al., 2018 |  |  |  |  |  |  |  |
| Oza, et al., 2023 |  |  |  |  |  |  |  |
| Pitocco, et al., 2013 |  |  |  |  |  |  |  |
| Sheikhy, et al., 2022 |  |  |  |  |  |  |  |
| Zhang, et al., 2021 |  |  |  |  |  |  |  |
|  | **Intention-to-treat crossover randomized controlled trials** | | | | | | |
| Khan, et al., 2006 |  |  |  |  |  |  |  |
| Yang, et al., 2020 |  |  |  |  |  |  |  |

**Appendix A.5. RoB 2 quality assessment graphs.** RoB 2 graphs for (**A** and **C**) intention-to-treat and (**B**) per protocol (**A** and **B**) parallel and (**C**) crossover randomized controlled trials.

**A)**

**B)**

**C)**

**Appendix A.6. Forest plots showing the BMI Z-score of adolescents.** Forest plots illustrating the meta-analysis results comparing BMI Z-score between adolescents with T1DM treated with a combination of metformin and insulin versus placebo plus insulin.

**Appendix A.7.** **Forest plots showing the TC level of adolescents.** Forest plots illustrating the meta-analysis results comparing TC level between adolescents with T1DM treated with a combination of metformin and insulin versus placebo plus insulin.

**Appendix A.8. Forest plots showing the TC level of adults.** Forest plots illustrating the meta-analysis results comparing TC level between adults with T1DM treated with a combination of metformin and insulin versus placebo plus insulin.

**Appendix A.9. Forest plots showing the TG level of adolescents.** Forest plots illustrating the meta-analysis results comparing TG level between adolescents with T1DM treated with a combination of metformin and insulin versus placebo plus insulin.

**Appendix A.10. Forest plots showing the TG level of adults.** Forest plots illustrating the meta-analysis results comparing TG level between adults with T1DM treated with a combination of metformin and insulin versus placebo plus insulin.

**Appendix A.11. Forest plots showing the LDL level of adolescents.** Forest plots illustrating the meta-analysis results comparing LDL level between adolescents with T1DM treated with a combination of metformin and insulin versus placebo plus insulin.

**Appendix A.12. Forest plots showing the LDL level of adults.** Forest plots illustrating the meta-analysis results comparing LDL level between adults with T1DM treated with a combination of metformin and insulin versus placebo plus insulin.

**Appendix A.13. Forest plots showing the HDL level of adolescents.** Forest plots illustrating the meta-analysis results comparing HDL level between adolescents with T1DM treated with a combination of metformin and insulin versus placebo plus insulin.

**Appendix A.14. Forest plots showing the HDL level of adults.** Forest plots illustrating the meta-analysis results comparing HDL level between adults with T1DM treated with a combination of metformin and insulin versus placebo plus insulin.

**Appendix A.15. Forest plot showing the DKA risk of adolescents.** Forest plot illustrating the meta-analysis results comparing the risk of DKA between adolescents with T1DM treated with a combination of metformin and insulin versus placebo plus insulin.

**Appendix A.16. Forest plot showing the DKA risk of adults.** Forest plot illustrating the meta-analysis results comparing the risk of DKA between adults with T1DM treated with a combination of metformin and insulin versus placebo plus insulin.

**Appendix A.17. Forest plot showing the HG risk of adolescents.** Forest plot illustrating the meta-analysis results comparing the risk of HG between adolescents with T1DM treated with a combination of metformin and insulin versus placebo plus insulin.

**Appendix A.18. Forest plot showing the HG risk of adults.** Forest plot illustrating the meta-analysis results comparing the risk of HG between adults with T1DM treated with a combination of metformin and insulin versus placebo plus insulin.

**Appendix A.19. Funnel plots of publication bias for** **the comparative efficacy.** Funnel plots of publication bias for the meta-analysis comparing (**A**, **a**) BMI, (**B**) BMI Z-score, (**C**, **c**) TIDD, and levels of (**D**, **d**) HbA1c, (**E**, **e**) TC, (**F**, **f**) TG, (**G**, **g**) LDL, and (**H**, **h**) HDL between (**A**, **B**, **C**, **D**, **E**, **F**, **G**, **H**) adolescents and (**a**, **c**, **d**, **e**, **f**, **g**, **h**) adults with T1DM treated with a combination of metformin and insulin versus placebo plus insulin.

**A)** **a)**

**B)**

**C)** **c)**

**D)** **d)**

**E) e)**


**F)** **f)**

**G)** **g)**


**H)** **h)**

**Appendix A.20. Funnel plots of publication bias for the comparative safety.** Forest plots of publication bias for the meta-analysis comparing the risk of (**A**, **a**) DKA, (**B**, **b**) GIAEs, and (**C**, **c**) HG between (**A**, **B**, **C**) adolescents and (**a**, **b**, **c**) adults with T1DM treated with a combination of metformin and insulin versus placebo plus insulin.

**A)** **a)**

**B)** **b)**


**C)** **c)**

**References**

[1] Amina R, Iftikhar A, Asher F, Fasiha F, Ruqaya N, Madiha S. Effect of Metformin as adjunct therapy with insulin in adolescent Pakistani people with type 1 diabetes. Pak J Pharm Sci. 2023;36(4):1085-8. doi: 10.36721/PJPS.2023.36.4.REG.1085-1088.1

[2] Anderson JJA, Couper JJ, Giles LC, Leggett CE, Gent R, Coppin B, et al. Effect of Metformin on Vascular Function in Children With Type 1 Diabetes: A 12-Month Randomized Controlled Trial. J Clin Endocrinol Metab. 2017;102(12):4448-56. doi: 10.1210/jc.2017-00781

[3] Bjornstad P, Schäfer M, Truong U, Cree-Green M, Pyle L, Baumgartner A, et al. Metformin improves insulin sensitivity and vascular health in youth with type 1 diabetes mellitus: Randomized controlled trial. Circulation. 2018;138(25):2895-907. doi: 10.1161/CIRCULATIONAHA.118.035525

[4] Burchardt P, Zawada A, Kaczmarek J, Marcinkaniec J, Wysocki H, Wierusz-Wysocka B, et al. Association between adjunctive metformin therapy in young type 1 diabetes patients with excess body fat and reduction of carotid intima-media thickness. Pol Arch Med Wewn. 2016;126(7):514-20. doi: 10.20452/pamw.3527

[5] Burchardt P, Zawada A, Tabaczewski P, Naskręt D, Kaczmarek J, Marcinkaniec J, et al. Metformin added to intensive insulin therapy reduces plasma levels of glycated but not oxidized low‑density lipoprotein in young patients with type 1 diabetes and obesity in comparison with insulin alone: a pilot study. Polskie Archiwum Medycyny Wewnetrznej. 2013;123(10):526‐32. doi: 10.20452/pamw.1925

[6] Codner E, Iñíguez G, López P, Mujica V, Eyzaguirre FC, Asenjo S, et al. Metformin for the treatment of hyperandrogenism in adolescents with type 1 diabetes mellitus. Horm Res Paediatr. 2013;80(5):343-9. doi: 10.1159/000355513

[7] Cree-Green M, Bergman BC, Cengiz E, Fox LA, Hannon TS, Miller K, et al. Metformin Improves Peripheral Insulin Sensitivity in Youth With Type 1 Diabetes. Journal of clinical endocrinology and metabolism. 2019;104(8):3265‐78. doi: 10.1210/jc.2019-00129

[8] Elbarbary NS, Ismail EAR, Ghallab MA. Effect of metformin as an add-on therapy on neuregulin-4 levels and vascular-related complications in adolescents with type 1 diabetes: A randomized controlled trial. Diabetes Res Clin Pract. 2022;186:109857. doi: 10.1016/j.diabres.2022.109857

[9] Gourgari E, Nadeau KJ, Pyle L, Playford MP, Ma J, Mehta NN, et al. Effect of metformin on the high-density lipoprotein proteome in youth with type 1 diabetes. Endocrinol Diabetes Metab. 2021;4(3):e00261. doi: 10.1002/edm2.261

[10] Hamilton J, Cummings E, Zdravkovic V, Finegood D, Daneman D. Metformin as an adjunct therapy in adolescents with type 1 diabetes and insulin resistance: a randomized controlled trial. Diabetes Care. 2003;26(1):138-43. doi: 10.2337/diacare.26.1.138

[11] Jacobsen IB, Henriksen JE, Beck-Nielsen H. The effect of metformin in overweight patients with type 1 diabetes and poor metabolic control. Basic Clin Pharmacol Toxicol. 2009;105(3):145-9. doi: 10.1111/j.1742-7843.2009.00380.x

[12] Janić M, Janež A, Šabović M, El-Tanani M, Rangraze I, Rizzo M, et al. Glucometabolic Efficacy of the Empagliflozin/Metformin Combination in People with Type 1 Diabetes and Increased Cardiovascular Risk: A Sub-Analysis of a Pilot Randomized Controlled Trial. Journal of Clinical Medicine. 2024;13(22):6860. doi: 10.3390/jcm13226860

[13] Khan AS, McLoughney CR, Ahmed AB. The effect of metformin on blood glucose control in overweight patients with Type 1 diabetes. Diabet Med. 2006;23(10):1079-84. doi: 10.1111/j.1464-5491.2006.01966.x

[14] Libman IM, Miller KM, DiMeglio LA, Bethin KE, Katz ML, Shah A, et al. Effect of metformin added to insulin on glycemic control among overweight/obese adolescents with type 1 diabetes a randomized clinical trial. JAMA - Journal of the American Medical Association. 2015;314(21):2241-50. doi: 10.1001/jama.2015.16174

[15] Lund SS, Tarnow L, Astrup AS, Hovind P, Jacobsen PK, Alibegovic AC, et al. Effect of adjunct metformin treatment in patients with type-1 diabetes and persistent inadequate glycaemic control. A randomized study. PloS one. 2008;3(10):e3363. doi: 10.1371/journal.pone.0003363

[16] Lund SS, Tarnow L, Astrup AS, Hovind P, Jacobsen PK, Alibegovic AC, et al. Effect of adjunct metformin treatment on levels of plasma lipids in patients with type 1 diabetes. Diabetes, obesity & metabolism. 2009;11(10):966‐77. doi: 10.1111/j.1463-1326.2009.01079.x

[17] Lunder M, Janić M, Japelj M, Juretič A, Janež A, Šabovič M. Empagliflozin on top of metformin treatment improves arterial function in patients with type 1 diabetes mellitus Clinical trial registration NCT03639545 NCT. Cardiovascular Diabetology. 2018;17(1). doi: 10.1186/s12933-018-0797-6

[18] Meyer L, Bohme P, Delbachian I, Lehert P, Cugnardey N, Drouin P, et al. The benefits of metformin therapy during continuous subcutaneous insulin infusion treatment of type 1 diabetic patients. Diabetes Care. 2002;25(12):2153-8. doi: 10.2337/diacare.25.12.2153

[19] Mondkar S, Khandagale S, Shah N, Khadilkar A, Oza C, Bhor S, et al. Effect of metformin adjunct therapy on cardiometabolic parameters in Indian adolescents with type 1 diabetes: a randomized controlled trial. Front Clin Diabetes Healthc. 2024;5:1353279. doi: 10.3389/fcdhc.2024.1353279

[20] Nadeau KJ, Chow K, Alam S, Lindquist K, Campbell S, McFann K, et al. Effects of low dose metformin in adolescents with type I diabetes mellitus: a randomized, double-blinded placebo-controlled study. Pediatr Diabetes. 2015;16(3):196-203. doi: 10.1111/pedi.12140

[21] Nwosu BU, Maranda L, Cullen K, Greenman L, Fleshman J, McShea N, et al. A Randomized, Double-Blind, Placebo-Controlled Trial of Adjunctive Metformin Therapy in Overweight/Obese Youth with Type 1 Diabetes. PLoS One. 2015;10(9):e0137525. doi: 10.1371/journal.pone.0137525

[22] Oza C, Mondkar S, Shah N, More C, Khadilkar V, Khadilkar A. A Pilot Study to Assess Effect of Metformin Therapy on Prevention of Double Diabetes in Indian Adolescents with Type-1 Diabetes. Indian J Endocrinol Metab. 2023;27(3):201-7. doi: 10.4103/ijem.ijem_46_23

[23] Petrie JR, Chaturvedi N, Ford I, Brouwers M, Greenlaw N, Tillin T, et al. Cardiovascular and metabolic effects of metformin in patients with type 1 diabetes (REMOVAL): a double-blind, randomised, placebo-controlled trial. The lancet Diabetes & endocrinology. 2017;5(8):597‐609. doi: 10.1016/S2213-8587(17)30194-8

[24] Pitocco D, Zaccardi F, Tarzia P, Milo M, Scavone G, Rizzo P, et al. Metformin improves endothelial function in type 1 diabetic subjects: a pilot, placebo-controlled randomized study. Diabetes, obesity & metabolism. 2013;15(5):427‐31. doi: 10.1111/dom.12041

[25] Särnblad S, Kroon M, Aman J. Metformin as additional therapy in adolescents with poorly controlled type 1 diabetes: randomised placebo-controlled trial with aspects on insulin sensitivity. Eur J Endocrinol. 2003;149(4):323-9. doi: 10.1530/eje.0.1490323

[26] Sheikhy A, Eydian Z, Fallahzadeh A, Shakiba M, Hajipour M, Alaei M, et al. Benefits of metformin add-on insulin therapy (MAIT) for HbA1c and lipid profile in adolescents with type 1 diabetes mellitus: preliminary report from a double-blinded, placebo-controlled, randomized clinical trial. J Pediatr Endocrinol Metab. 2022;35(4):505-10. doi: 10.1515/jpem-2021-0704

[27] Yang D, Yan J, Deng H, Yang X, Luo S, Zheng X, et al. Effects of Metformin Added to Insulin in Adolescents with Type 1 Diabetes: an Exploratory Crossover Randomized Trial. Journal of diabetes research. 2020;2020:7419345. doi: 10.1155/2020/7419345

[28] Zawada A, Naskręt D, Burchardt P, Niedźwiecki P, Piłaciński S, Wierusz-Wysocka B, et al. Metformin added to intensive insulin therapy improves metabolic control in patients with type 1 diabetes and excess body fat. Pol Arch Intern Med. 2018;128(5):294-300. doi: 10.20452/pamw.4241

[29] Zhang X, Xu D, Xu P, Yang S, Zhang Q, Wu Y, et al. Metformin improves glycemic variability in adults with type 1 diabetes mellitus: an open-label randomized control trial. Endocr Connect. 2021;10(9):1045-54. doi: 10.1530/EC-21-0146
